# Implementation considerations for non-communicable disease-related integration in primary health care: a rapid review of qualitative evidence

**DOI:** 10.1186/s12913-023-09151-x

**Published:** 2023-02-18

**Authors:** N. Leon, H. Xu

**Affiliations:** 1 Independent Public Health Researcher, Charlottesville, VA USA; 2 Department of Epidemiology, Brown University School of Public Health, Providence, RI USA; 3 South African Medical Research Council, Cape Town, South Africa; 4 Department of Noncommunicable Diseases, World Health Organization, Geneva, Switzerland

**Keywords:** Primary health care (PHC), PHC integration, Integrated service delivery, Noncommunicable diseases, Rapid review

## Abstract

**Background:**

Integrated delivery of primary health care (PHC) services is a health reform recommended for achieving ambitious targets of the Sustainable Development Goals and Universal Health Coverage, responding to growing challenges of managing non-communicable and multimorbidity. However, more evidence is needed on effective implementation of PHC integration in different country settings.

**Objective:**

This rapid review synthesized qualitative evidence on implementation factors affecting integration of non-communicable disease (NCD) into PHC, from the perspective of implementers. The review contributes evidence to inform the World Health Organizations’ guidance on integration of NCD control and prevention to strengthen health systems.

**Method:**

The review was guided by standard methods for conducting rapid systematic reviews. Data analysis was guided by the SURE and WHO health system building blocks frameworks. We used Confidence in the Evidence of Reviews of Qualitative Research (GRADE-CERQual) to assess the confidence of the main findings.

**Results:**

The review identified 81 records eligible for inclusion, from 595 records screened. We sampled 20 studies for analysis (including 3 from expert recommendations). Studies covered a wide range of countries (27 countries from 6 continents), the majority from low-and middle-income countries (LMICs), with a diverse set of NCD-related PHC integration combinations and implementation strategies. The main findings were categorised into three overarching themes and several sub-themes. These are, A: Policy alignment and governance, B: Health systems readiness, intervention compatibility and leadership, and C: Human resource management, development, and support. The three overarching findings were assessed as each having a moderate level of confidence.

**Conclusion:**

The review findings present insights on how health workers responses may be shaped by the complex interaction of individual, social, and organizational factors that may be specific to the context of the intervention, the importance of cross-cutting influences such as policy alignment, supportive leadership and health systems constraints, knowledge that can inform the development of future implementation strategies and implementation research.

**Supplementary Information:**

The online version contains supplementary material available at 10.1186/s12913-023-09151-x.

## Background

### Introduction

The global burden of non-communicable disease (NCD) is growing and becoming the leading cause of preventable mortality [1–3]. NCDs, including cardiovascular diseases, cancer, diabetes and chronic respiratory diseases, are now responsible for 74% of deaths worldwide, with the risk of dying prematurely from NCD being nearly double in low-and middle-income (LMIC) countries [3]. To address this growing burden of disease, the World Health Organization (WHO) has called for strengthening of health systems to address the NCD burden, “through people-centred primary health care and universal health coverage” [3], which includes integrated delivery of primary health care (PHC).

Primary health care (PHC) is defined by WHO as: “A whole-of-society approach to health intended to maximize the level and distribution of health and well-being through three components: (a) primary care and essential public health functions as the core of integrated health services; (b) multisectoral policy and action; and (c) empowered people and communities.” [4]. PHC therefore comprises essential health services that should be universally accessible for individuals, families, and communities, and that provides the first level of health care treatment and referral for acute and chronic disease, as well as disease prevention and health promotion services [5]. For the past four decades since the 1978 Alma Alta Declaration on PHC, integrated delivery of PHC services has been recommended as a health sector reform to provide more efficient and effective and equitable access to Universal Health Coverage (UHC) [5, 6], and more recently, to achieve the Sustainable Development Goals (SDGs) [7]. However, globally, the pace of implementation and impact of PHC integration has been variable, and hindered by a diverse set of factors, including limited political commitment, logistical barriers and health system constraints [6, 8, 9]. Given the complexity of integration reforms, it is recommended that programmes and policies on integrated service delivery consider context-specific factors related to health systems and populations [9, 10], and this should include a better understanding of the role of health-care workers who are at the forefront of implementation.

Integration of health service delivery is a health reform recommended for achieving ambitious targets set for controlling the rising burden of NCDs (including mental health disorders), and comorbidity of other disease in people living with long-term communicable disease, like HIV and TB [7, 11–14]. Against a background of constraints of resources, and the rise of NCDs and other disease priorities (such as HIV, TB and reproductive, maternal, child and adolescent health), integrated delivery of NCDs at primary care level is potentially a valuable tool in LMICs [11]. This is because in LMICs, underfunded health care systems may be further weakened by fragmented, uncoordinated services that risk being overburdened by the burgeoning NCD epidemic [8, 11, 15].

Integration requires various organizational changes in health systems to ensure connectivity, linkage, alignment, and collaboration in the delivery of various service, with view to ensure more efficient, effective, equitable health care for the complex burden of diseases [13, 15, 16]. Integration mechanisms might include changes to improve synergies in the functions of governance and leadership, financing, planning, service delivery, monitoring, evaluation, and generation of demand [13].

While the aim is for integrated services to create synergistic use of the available resources and reducing duplication, reconfiguration or reform of health services may face barriers [8, 13, 15, 16]. Challenges associated with integrating NCD with other PHC services, include problems related to supply chain, human resources, referral systems, staff attitudes and skills, stigmatization, health information systems, and monitoring and evaluation [11, 14]. There is no standard definition of integration—integration may take different forms and be influenced by different country level factors, including the country’s disease burden and health system configuration [8, 11, 16].

Health-care workers, managers and policymakers are key stakeholders in implementation of PHC integration reform and can influence successful implementation and impact. Health-care worker response may be influenced by their experience and perceptions of individual, organizational and system level factors [8, 17–20]. For instance, health workers in low resource settings may be struggling with issues such as chronic staff shortages, multiple demands, and poor performance management [8, 10, 20]. In high resource settings, organizational factors such as high levels of health worker specialization and restrictive financial mechanisms may shape staff responses to integration of services [8]. Health-care workers who are informed and engaged may act in a manner that supports the policy expectations for the new reform initiative [18–20]. Health workers not feeling engaged and supported may apply their discretion in ways that give them more control to manage the stress of the work, but that does not does not necessarily support effective implementation [17, 19, 20]. For instance, workers may act to reduce the complexity of the work, or to conserve resources by rationing services, or allocating their own benefits and sanctions to patients, in ways not intended by the policy reform [17, 19, 20]*.* Questions remain on how best to provide integrated PHC services, and more evidence is needed on feasibility, acceptability, and effectiveness of PHC integration types in various settings, including what are health worker perspectives of implementation considerations [11, 14–16, 21, 22].

This is a rapid review to synthesize qualitative evidence on factors influencing the implementation of NCD-related integration at primary care, from the perspectives of health care providers, managers, and policymakers. The rapid review was commissioned as one source of evidence to inform WHO guidance on integrating the prevention and control of noncommunicable diseases into national responses to HIV/AIDS, tuberculosis and reproductive health to strengthen health systems.

## Method

### Design

We were guided by rapid review methods, which is a simplified approach to systematic review approach for synthesizing qualitative evidence, to produce findings within a short time. A rapid review is defined as “a type of knowledge synthesis for which the steps of the systematic review are streamlined or accelerated to produce evidence in a shortened timeframe.” [23]. This rapid review is embedded in a larger, ongoing EPOC Cochrane qualitative evidence synthesis (QES) project, titled, “Healthcare workers' perceptions and experience of primary healthcare integration: a qualitative evidence synthesis” (referred to here as the ‘parent review’). The primary objective of the parent review was: “To identify, appraise, and synthesise qualitative studies exploring healthcare workers' perceptions and experiences of PHC integration; to identify the factors influencing healthcare workers' perceptions and experiences of PHC integration interventions” [24]. Details on the parent review methodology can be found in the published review protocol [24]. The rapid review drew on the literature search conducted for the parent review. This was for pragmatic reasons of feasibility, and we determined there was sufficient overlap between the Cochrane parent review and the WHO rapid review question.

Our rapid review was guided by the rapid review recommendations for streamlining methods used for systematic reviews, including limiting the number of electronic data bases for searching, using dual or single reviewers for the stages of abstract screening, full text reviewing, data extraction and synthesis, and for assessing the quality of studies [23]. We built in additional quality assurance steps as recommended by the WHO practical guide for rapid reviews to strengthen health policy and systems [23]. We summarised our rapid review streamlined approaches, quality assurance steps, as well as deviations from standard recommendations in Table 1. Deviations were due to pragmatic and logistical reasons of feasibility and time and include drawing on a pre-existing set of search records, creating a smaller, more manageable sampling frame before final sampling, sourcing additional records from experts, and doing quality appraisal on only the selected studies. We reflect on these and other study limitations in the Discussion section. We provide a completed PRISMA 2020 checklist to illustrate compatibility with review procedures and reporting guidelines (See Additional file 1).

**Table 1:** Summary of rapid review streamlined approaches

Steps and stages	Review characteristics	Streamlining and quality assurance steps
Duration of review	10 weeks	An external, independent researcher consultant contracted by WHO to produce a technical report to inform their WHO integration guidance development process. Assisted by an internal, senior WHO staff member
Embedding the review search	Accessed the large database of an ongoing Cochrane systematic review, with a published review protocol	This was a pragmatic decision to capitalize on synergies with an ongoing review. This could be considered an adaptation of the standard rapid review search strategy that allows for searching smaller number of electronic data bases
Protocol	Rapid review protocol was developed	Review protocol developed by two authors and presented during WHO working group meeting
Author details	Two reviewers, with the external consultant as lead reviewer	Lead author, qualitative health systems researcher, senior author of parent qualitative systematic review protocol Co-author, content expert on integrated service delivery and WHO technical lead, evidenced based medicine and health system development background
Creating a data base for the rapid review	Literature search and search strategy within the parent records data base	Used existing database of records from the parent review. Used a set of terms related to the health programmes of interest, to search the titles and abstracts and identify articles for the rapid review database. The was done by one author (NL)
Study selection	Title and abstract screening	Adapted the inclusion and exclusion criteria of the parent review to our research question and used this for title and abstract screening. Title and abstract screening by two authors screening each record independently so each record was screened twice. Inter-rater ability was developed through both reviewing a small number of papers at the start (*N* = 10). Where there were disagreements, we reached consensus through discussion
	Full text review of 120 eligible studies	Full text review by one author (NL), with co-author doing random checks for agreement
	Studies selected for inclusion = 81. Sampling was done using a combination of purposive and variation sampling. Sampling was done in two stages, starting with creating a smaller, more manageable sampling frame of 51 out of 81 studies and then sampling 17 out of 51 studies in the sampling frame	The sampling approach was done by both authors, through discussion of each sampling choice The two-stage sample approach is a deviation from the standard streamlining recommendation for rapid reviews. While this was done for pragmatic reasons of time, it is considered a limitation as it could have skewed the sample For context and additional information, provide a table that describes the characteristics of the other 34 studies in the sampling frame (See Additional file 4)
	Selection of additional studies	We sourced studies from WHO expert to address geographical gaps This was done for pragmatic and equity reasons but could be considered a deviation from standard rapid review practice as it could have skewed the sample
	Quality appraisal of included studies, using an adjusted version of the CASP tool	During the first sampling phase we did an informal, quick appraisal of study quality with the aim of including with richer qualitative data in the sampling frame. We only did a formal quality appraisal of the 20 included studies, and not of the other 34 studies in the sampling frame
Data extraction	Developed an Excel data extraction template. Used a combination of SURE framework and WHO health system building blocks to guide the categories of information extraction	Data extraction template was piloted and adjusted accordingly Data extraction was done by 1^st^ author (NL) and checked by 2^nd^ author (HX)
Data synthesis	We provide a narrative synthesis of key findings, categorised into three overarching themes, and sub-themes	Data was synthesised within and across studies, using the categories in the data extraction template. Key themes and sub-themes emerged, and these were presented in narrative form, supported by referencing contributing studies, and illustrated with quotes
	We summarised the key themes into three high-level, overarching findings. We then applied the CERQual tool. This provides an assessment of the level of confidence we have that the finding represents the underlying data that contributed to the finding	We provided a CERQual assessment of the 3 key findings. This is not a requirement for a rapid review, and we included this as additional tool to help inform the guidance development team

### Searching databases

A systematic search for literature for the parent review was performed in February 2020, with an additional search on 29 July 2020, using a search strategy designed by the EPOC Cochrane information specialist. (See Additional file 2 for details of the search terms and strategy). The parent search used a MEDLINE search strategy to search PDQ-Evidence, Epistemonikos Foundation for related reviews to identify eligible studies for inclusion (www.pdqevidence.org/) and the following electronic databases: MEDLINE Ovid, CINAHL EBSCO, Scopus Elsevier, Global Index Medicus, World Health Organization (WHO), ProQuest, Dissertations and Theses Global databases. We limited the rapid review records to 2010, guided by the assumption that NCD integration programmes were less common prior to this date. The United National General Assembly resolutions to integrate NCD prevention control in primary health care was made in 2011 [25]. A scoping review of integrated care for multimorbidity and NCD diseases found that most programmes that applied integrated models and programmes were published after 2010 [26].

We identified geographical gaps in the list of selected studies (Latin America, East Asia, Francophone Africa, and fragile state settings). Fragile state settings mean countries or situations with challenges that have resulted from fragility and conflict, which is an important equity consideration for WHO. We used a pragmatic approach to attempt to fill these gaps since the WHO has extensive experience of implementing and adapting NCDs prevention and control in LMICs. This involved asking experts in WHO regional networks to suggest literature, including searching African Index Medicus library database hosted by WHO Regional Office for Africa for literatures from African francophone countries.

### Inclusion and exclusion criteria

#### Inclusion criteria

The criteria for inclusion and exclusion were guided by the SPIDER question formulation tool and are detailed in a selection criteria guide. (See Additional File 3 for a detailed guide on inclusion and exclusion criteria for screening records for the parent review, which we adapted for the rapid review).

#### *S*ample

Health workers in primary care (health policy makers, managers, frontline workers), general professional PHC workers, including community health care workers (CHWs). The geographical scope is global (countries of all income-levels and geographical distribution).

#### Phenomenon of Interest

Integration of NCDs (multiple NCDs with each other, NCDs plus communicable diseases and reproductive, maternal, child and adolescent health in primary care settings, and general PHC integration), with focus on PHC level as the site of delivery of care. Public, public–private partnerships, and private institutions (where private institutions were assessed as providing public sector primary care on behalf of the government).

#### Design

Qualitative and mixed method studies. Mixed-methods studies are eligible only if qualitative data can be extracted. This includes some evaluation and implementation studies.

#### Evaluation

Perceptions and experiences of implementation considerations for NCD integration (barriers and facilitators, recommendations for implementation).

#### Research type

Qualitative and mixed method studies.

#### Exclusion criteria

We excluded specialist, non-PHC level health workers, integration services not linked to PHC facility as the sight for delivering care, multi-sectoral integration where the focus was beyond health, and children’s health services.

### Study selection

**Fig. 1:**
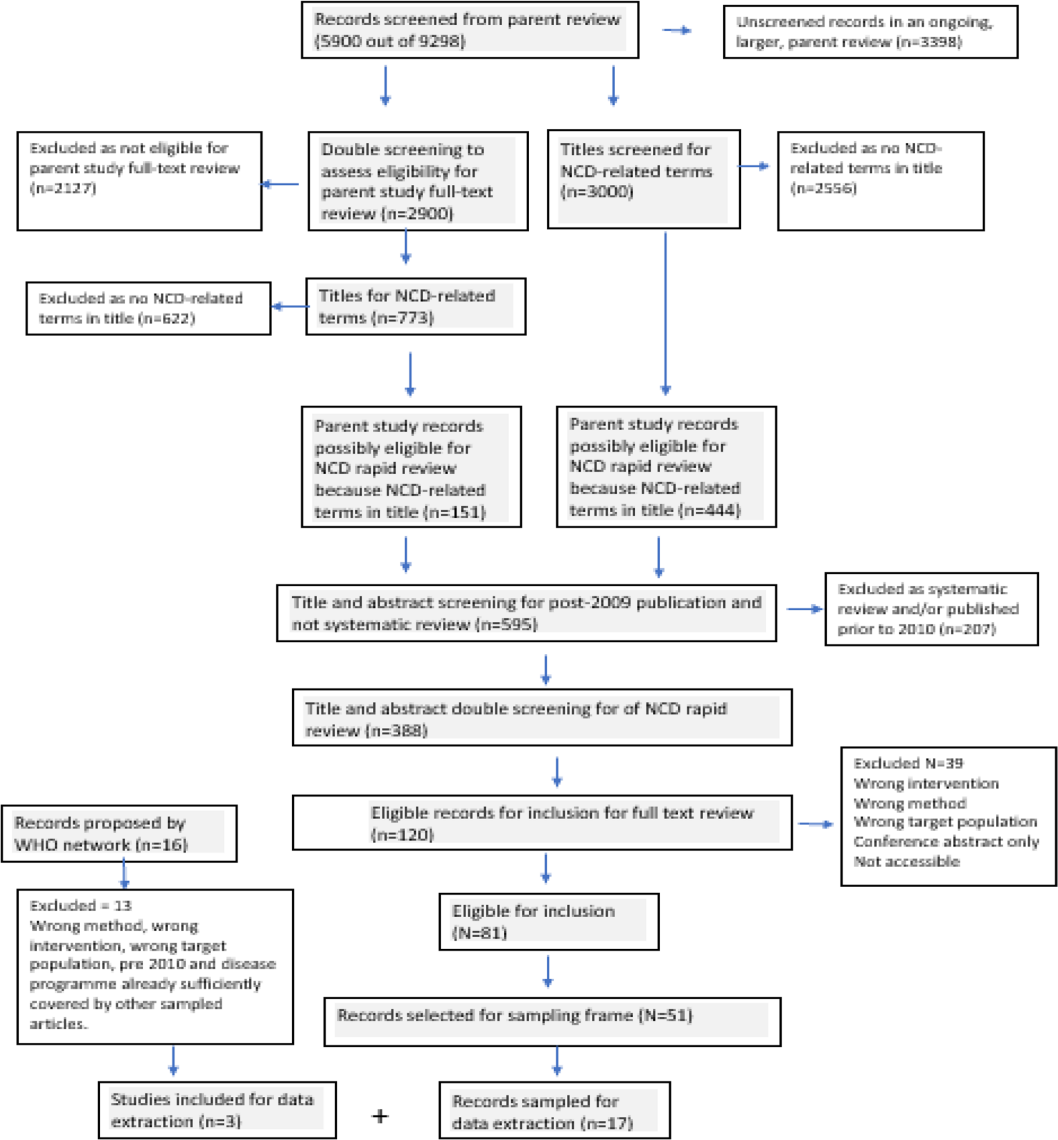
PRISMA diagram of data flow for WHO NCD-PHC integration rapid review

In the main search for the parent review, 9289 records were uploaded to Covidence, an electronic software tool, for managing the screening process (https://www.covidence.org). The Fig. 1, PRISMA diagram illustrates the process for selecting studies. By 13 June 2020, the parent review team had identified 773 eligible records for full text screening for the parent review (out of approximately 3000 records) using independent screening by two reviewers. Reviewers for the parent review received extensive training to ensure interrater reliability and conflicts were resolved by a third reviewer. In Covidence, we filtered all records that contained a reference in the title that was potentially relevant to our NCD rapid review, including the following terms: noncommunicable disease, communicable disease, chronic disease, HIV, TB, mental health, maternal and reproductive health. Of the 773 records, 151 records were considered potentially eligible for the NCD rapid review through this filtering. To expand our database, the lead author screened an additional 3000 records in the parent review (single screening, not using two independent screeners), which identified another 444 potentially eligible NCD-related records. Thus 595 records (151 + 444) were identified for initial screening for the rapid review, and after removal of studies prior to 2010 and systematic reviews, 388 records were identified for further screening for the rapid review. Using an Endnote library, and the inclusion guideline criteria, the abstracts of 388 records were screened independently by two reviewers (NL and HX). We first practiced on a set of 10 papers to ensure a standard approach and mismatches were resolved by consensus between the two screeners. A set of 120 records were identified by both reviewers as eligible for full text screening. Full-text screening was done by a single reviewer (NL), with spot checks done by the 2^nd^ reviewer, and we identified 81 eligible studies for inclusion. We also reviewed the full texts of 16 papers suggested through WHO sources and selected 3 for inclusion and data extraction.

### Sampling

When a large number of studies are available for synthesizing qualitative evidence, all cannot usually be appraised rigorously or in-depth, and sampling is required [27]. To sample studies for data extraction, we first designed sampling criteria to guide a combination of purposive and maximum variation sampling. Purposive sampling was conducted for a good fit with our inclusion criteria, especially geographical variation, NCD-related interventions (and/or more general PHC integration), and integration strategy (e.g., integrating previously vertical disease programmes, care coordination, multidisciplinary teams, integrated health system functions mechanisms), and differentiated care level (whether PHC only or across PHC, hospital and community-based services). We also sought a balance between scope and breadth of the studies (for example, favoring studies that covered multiple countries in one study), the integration focus of the study (disease programme-specific focus versus transversal health system focus), and depth of findings (such as where there is detailed qualitative findings presented that are well illustrated with quotes and extracts). We did the sampling in two stages, first selecting a smaller sampling frame (51 out of 81 studies), then sampling 17 (out of the 51 studies). First, we applied the sampling criteria loosely to the 81 studies in a quick overview, to select a smaller, more manageable number of studies for a sampling frame. For example, in the first stage we focused more on getting geographic representation within the sampling frame. The 51 studies in the sampling frame (out of 81 identified for inclusion), were selected by applying the sampling criteria in an iterative fashion; this involved moving back and forth between the studies that were being sampled and the full set of 81 studies, to keep adding to the sampling frame, based on the other sampling criteria. We then used maximum variation sampling to select a smaller, more manageable sample of 17 studies for data extraction. We applied a similar iterative process of moving back and forth between the studies sampled and the sampling frame, and swopping some out, to ensure the studies in the final sample covered the criteria we were interested in. To address geographical gaps, we supplemented this with 3 purposively selected studies from 16 sources suggested by WHO colleagues. The characteristics of the sampled studies are included in Table 2 and in Additional file 4, we present a table that describe the characteristics of the rest of the 34 studies in the sampling frame (author, date, county, method, and study aim).

**Table 2:** Description of characteristics of included studies and CASP assessment

**Author, year**	**Study method and data collection**	**Country**	**Diseases, mechanisms of integration**	**Intervention description**	**CASP quality assessment**
Duffy, 2017	Qualitative. Key informant interviews and literature reviews	Zambia, Kenya, Uganda, and Cambodia	NCD and HIV services integration	Three different intervention models of integrating HIV and NCD services in LMICs	**Minor concerns** **Partly due to context, sampling, method & analysis**
Fuller, 2015	Qualitative: key informant interviews, focus groups and literature review	Australia	NCD integration into PHC: Creating a new cadre of nurses for coordination of chronic care in general practices	The South Australian Initiative General Practice (GP) Plus Practice Nurse Initiative (2007–2010) to establish what is needed to support the development of the NCD chronic management role of practice nurse. Designed to build Chronic Disease Management (CDM) capacity in General Practice, through the employment and support of practice nurses as care coordinators, who would establish chronic disease management systems	**No to minor concerns**
Kalonji, 2019	Qualitative. Document reviews	South Africa	Integration of previously vertical HIV and TB services	HIV and TB services are provided by the same trained healthcare provider at the same visit, as a ‘one-stop service at facility and community level	**Moderate to serious concerns** **Limited qualitative data**
Kozlowska, 2020	Qualitative. Document reviews and key informant interviews	United Kingdom	NCD: Coordination of diabetes care across different health care organizations	Integration in the delivery of diabetes care across primary, community and specialist care, through commissioning collaborative care between service organizations, and driven by a transformation team	**No or very minor concerns**
Lawn, 2014	Qualitative. Ethnographic observation and focus groups	Australia	PHC care coordination across health and social service organizations through co-location in a single purpose-built center	Integration of PHC services by co-locating health and social service delivery organizations side by side in a purpose-built GP Plus center Including PHC, mental health, dentistry, allied health, pathology, and youth services and visiting services, to provide coordinated care	**No concerns**
Lovero, 2019	Mixed-methods: quantitative and qualitative. Key information interviews	South Africa	TB & maternal health integration by introducing new screening and referral mechanisms	The most common practices were 1) mental health screening by nurses and 2) mental health care referrals to be made to mental health practitioners	**Moderate concerns** **Limited amount and thin quality data**
Lupafya, 2016	Mixed methods. Semi-structured questionnaires were used to collect quantitative and qualitative data	Malawi	NCD	Some of the planned interventions recommended by the Malawi NCD Action Plan (2012–2016), mainly awareness raising and improvement of clinical management of NCDs	**Moderate concerns** **Limited qualitative data and analysis**
Petersen, 2019	Qualitative. Key informant interviews	South Africa	Mental health service integration into PHC	Using a range of strategies for integration of mental health service and systems, across the WHO building blocks, across the PHC and community-based levels, adapted to country setting. Including: strengthening governance structures, district financing, training, specialist supervision, technical support information and drug supply systems, monitoring and evaluation	**No concerns**
Schmidt, 2016	Qualitative. Document review and qualitative interviews	Australia	NCD: New cadre of worker and teamwork, for NCD care-coordination	This involved teamwork between member of the PHC and community-based team, including professional support for Indigenous Health Workers (IHWs)-led case management. “Examples may include a pharmacist performing home medication reviews to improve medication adherence and appointments with a diabetes educator to promote lifestyle changes and client capacity for self-management." (pg. 2)	**No to minor concerns**
Shelly, 2019	Qualitative. Key informant interviews	Tanzania	Integration of previously vertical HIV and maternal, newborn, and child health (MNCH) through extending CHW roles.	Community health workers (CHWs) working with HIV positive patients were trained to take on a dual role with additional duties to increase antenatal care (ANC) utilization and health facility deliveries, with supervision from a single HIV health care worker	**Minor concerns**
Venables, 2016	Mixed methods. Key informant interviews, focus groups and observation	Kenya	NCD and HIV: Task- shifting, medication dispensing to community clubs	Integration of HIV and NCD medication dispensing at community-based Medication Adherence Clubs (MACs), for stable patients to collect their medicine every three months through a club, rather than through individual clinic appointments. (pg.1)	**None or very minor concerns**
White, 2013	Mixed methods. Key informant interviews and focus groups	Cambodia	HIV and reproductive health integration	The Linked Response intervention was piloted “to promote pregnant women’s HIV testing and general utilization of reproductive health facilities as well as improve the follow-up of HIV-positive women and exposed infants through strengthened referral and operational linkages amongst health facilities/services and community-based support interventions for PLHIV." (pg. 1)	**No to minor concerns**
Mayhew, 2017	Qualitative: Case studies using document reviews	Kenya	HIV and Reproductive health care coordination, using a range of health systems strengthening strategies	“The Integration Initiative. (Integra)…is the largest evaluation trial of integrated HIV and RH services globally” (g2). In 2009- 2011, in Kenya and Swaziland, Integra evaluated different models of integrating HIV services into family planning, STI and cervical cancers screening, through provision of training, equipment and supplies, and supportive supervision by nurse mentors	**No concerns**
Stadnick, 2019	Qualitative. Comparative case studies drawing on key informant interviews	Israel, Nigeria, Peru, UK, USA, Vietnam	PHC general integration of services. Multiple mechanisms; care coordination, governance, funding mechanisms, multi-disciplinary teams	Multiple interventions: Integrated care of older adults, depression screening and treatment in PHC, continuity of care model to reduce pre-terms birth, treatment for autism, insurance reform for integrated mental health care, patient -centered medical home models	**No to minor concerns**
Steele Gray, 2018	Qualitative. Multiple case studies drawing on key informant interviews and document reviews	Canada and New Zealand	NCD Care co-ordination and quality improvement, using ICT support	ICT support elements aligned to the Chronic Disease Model activities for older adults with complex needs and those with and multimorbidity. Includes self-management, multi-disciplinary teamwork, and decision-support. This included ICT provision for electronic health records (EHRS), mHealth, Telehealth, online resources/systems that support quality improvement and patient engagement; electronic clinical guidelines, training for providers and monitoring and evaluation	**No to minor**
McIntosh, 2018	Qualitative. Multiple case studies drawing on key information interviews, focus groups and literature review	Germany, Greece, Italy, Northern Ireland, Portland, Portugal, Scotland, Spain, Sweden	PHC integration of pharmacy services. Polypharmacy management, care coordination, and supportive policy and legislation	"Common themes regarding the development and implementation of polypharmacy management initiatives were locally adapted solutions, organizational culture supporting innovation and teamwork, adequate workforce training, multi-disciplinary teams, changes in workflow, redefinition of roles and responsibilities of professionals, policies and legislation supporting the initiative, and data management and information and communication systems to assist development and implementation." (pg. 2)	**No concerns**
Ignatowicz, 2014	Qualitative. Key informant interviews, focus groups, observation, literature review	United Kingdom	PHC integration through financial and governance arrangements, quality of care improvements, alignment of information & ICT systems and funding model incentives	"The Northwest London ICP is a large-scale innovative program linking nearly 100 general practices, three community service providers, two mental health providers, two acute providers, and five local authorities.” “ICP is grounded in five principles: invest to save, shift care from acute care to community, raise the quality of patient care, population-based approach, and alignment of incentives and information and governance." [2, 3]	**No concerns**
Montenegro, 2011	Qualitative. Multiple case studies drawing on expert meetings, country consultations, document, and literature reviews	Argentina, Belize, Brazil, Chile, Cuba, Ecuador, Mexico, Paraguay, Trinidad and Tobago, and Uruguay, Central America, and Eastern Caribbean	PHC general, emergency care, maternal, child, reproductive health & social support services	Integrated Health Service Delivery Networks (IHSDNs) Multiple mechanism, from governance and policy reform, organisational co-ordination through networks, integration of previously vertical programs, integration of social support services with health care Common elements of the IHSDN interventions were, “Models of care, Governance and strategy, Organization and Management: Financial allocation and incentives	**Moderate concerns. Limited qualitative data**
Hosey, 2016	Mixed methods. Case study approach drawing on document reviews	Six US-Associated Pacific Islands	NCD and TB: Diabetes and TB integration via multi-disciplinary teams	NCD Collaborative model that proactively targets health system change and expands population outreach efforts. Interventions included clinic redesigns (new clinic days or one-stop shop), multi-disciplinary team approach, increased access, and quality of services, and use of a Chronic Disease Management System (CDEMS) (pg. 6)	**Moderate concerns. Due to concern about methods**
Zou, 2020	Qualitative study. Process evaluation drawing on qualitative interviews and observation	China	NCD: Integration of new preventive services into existing NCD services, at differentiated levels of care	A comprehensive intervention to improve cardiovascular disease (CVD) in patients with hypertension, diabetes, and high CVD risk. “Major initial and refresher trainings for providers, a clinical and prescribing guide, health education, and adherence support. Patients met with their family doctors monthly. Treatment supporters were selected along with patients to encourage adherence to drugs and lifestyle changes [9]." (pg. 2)	**No concerns**

### Data extraction and quality appraisal

Data were extracted and synthesized by the lead author (NL) and checked by the co-author (HX). The quality of studies in the final sample was assessed with and adapted version of the Critical Appraisal Skills Programme (CASP) tool for assessing quality of qualitative studies [28, 29], to identify methodological limitations to be considered in appraising confidence of findings of the review.

Regarding quality assessment of studies using CASP, all the selected studies included a clear statement of aim, research methods, and data sources appropriate for answering the research question. Most studies had sufficient rigor in data analysis, clearly stated findings, and reflections on the strength and limitations of the study. Most studies were therefore rated as having no or minor quality concerns. For studies with moderate and moderate-to-serious concerns, this was mainly due to the limited amount and depth of qualitative findings. Table 2 provides a description of the characteristics of the studies, including, author, date, title, country, study methods and data collection, disease and mechanisms integrated, description of the intervention and the overall CASP quality assessment. The core content relevant to the research question was extracted onto a Excel sheet, guided by categories of the ‘Supporting the Use of Research Evidence’ (SURE) framework (a framework on factors influencing implementation of health interventions) [30]. We used the WHO Health systems building block framework to guide further synthesis of data [31]. The items in the data extraction template was piloted and adjusted to expand and amalgamate categories. Categories of information extracted were knowledge, skills, attitudes, and motivation for change of decision-makers and implementers; broader health system factors and determinants (leadership, management, supervision, communication, change management and organizational culture); the quality and compatibility of the intervention; support services (human resources, finances, infrastructure, supply systems, health information and monitoring systems); and community and patient participation. Contextual information on broader political, legislative, and regulatory environment was also extracted.

### Data analysis and synthesis

The authors used the data matrix sheet to read, review and summarize the data from all studies into each of the SURE categories to identify common and divergent points within categories and common themes. For each SURE category (for example, provider knowledge and skills), data were transferred from the matrix sheet into a Word document and then synthesized into draft findings. In identifying themes, some SURE categories were combined (for example, motivation for change combined with the communications and change management), and new sub-categories emerged (for example, alignment of policy with resources, alignment of leadership at different levels and transformative leadership). We identified three main findings with some overlap between the categories in the SURE framework and the WHO health system building blocks and considered additional categories emerging from the data. The findings are presented in narrative form, including outlining of sub-categories of findings, as is appropriate for qualitative synthesis, and components of findings are referenced with the studies that contributed to the finding. For the Summary of findings (SOF) table we condensed the 3 overarching findings and present three highly aggregated findings (see Table 3: Summary of findings and confidence levels).

**Table 3:** Summary of findings and confidence levels

**Theme**	**Finding**	**Data source**	**Confidence level**
A: Policy alignment and governance	Appropriate governance (political, legal, and administrative), that includes an enabling policy environment and policy alignment across management levels, as well as alignment with health system support services, are key to enable implementation and for sustainable integration reforms	[14, 32–48]	**Moderate confidence** – it is likely that the review finding is a reasonable representation of the phenomenon of interest. (Downgraded due to minor concerns re methodological limitations, relevance, and adequacy)
B: Health systems readiness, intervention compatibility and leadership	Health system readiness for change, compatibility of the new integration intervention with existing organisational practices, and the quality of leadership are key factors that can influence the success of integration	[14, 32–50]	**Moderate confidence**—it is likely that the review finding is a reasonable representation of the phenomenon of interest (Downgraded due to minor concerns re methodological limitations, relevance, and very minor concerns re adequacy)
C: Human resource management, development and support	Human resource management, development and support are major considerations for implementing integration of services, with the need for strategic realignment of financial, management, and human resource and supervision systems, in support of the integration reform objectives. This involves human resource availability, appropriate skills, and training of staff, as well as supportive management and supervision	[14, 32–37, 39–41, 44, 45, 47–50]	**Moderate confidence** – it is likely that the review finding is a reasonable representation of the phenomenon of interest. (Downgraded due to minor concerns re methodological limitations, relevance, and adequacy)

The lead author (NL) is a qualitative health systems researcher with experience in systematic review methodology and the co-author is a WHO content expert on health service integration and she is the lead technical officer for the WHO guidance on integrated service delivery for NCDs.

### Appraising the confidence of the evidence

To support the use of the findings in decision-making, we applied the GRADE-CERQual approach [51] for a rapid assessment of the confidence of each review finding, focused on the three highly synthesized findings. This assesses the extent to which a review finding can be considered a reasonable representation of the phenomenon of interest. We assessed four components of confidence: methodological limitations of underlying studies, coherence (fit between the finding and the underlying data), adequacy (amount and degree of richness of data sources) and relevance (the extent of the applicability of the underlying evidence to the review question).

Ethical approval.

The review does not require ethical approval as it is a synthesis of published papers.

## Results

### Study description

Twenty studies published between 2011 and 2020 were used in this synthesis (See Table 2 for study details). The studies were conducted in 27 countries in Africa, the Americas, Asia, Europe, and Oceania. Some studies included several countries. African countries were represented either individually or with other countries: Kenya [14, 32, 33], Nigeria [34], South Africa [35, 36], United Republic of Tanzania [49], Malawi [37], Uganda [14, 36], Ethiopia [36] and Zambia [14]. Studies in the Americas covered seven Latin American countries (Brazil, Chile, Colombia, Dominion Republic, Guatemala, Peru and Trinidad and Tobago) [38]; Canada [39] and the USA [34] in North America and six US-administered Pacific islands [40]. The Asian countries studied were Cambodia [14, 50], China [41], India [36] and Israel in the Middle East [34]. Case studies in European countries comprised three in the United Kingdom [34, 42, 43] and one each in five other countries (Germany, Northern Ireland, Scotland, Spain (Catalonia) and Sweden) [44]. Oceania was represented by Australia [45–47], New Zealand [39], and US-administered Pacific Islands [40]. Most countries were from LMIC settings, and the remaining nine were high-income countries. Most studies addressed integration of NCDs, either with other NCDs [37, 39, 40, 43–45, 47] or with HIV or maternal, child and reproductive health [14, 33] or mental health [35, 36]. Others addressed integration of HIV with other disease programmes, such as maternal, child and reproductive health and TB [32, 48–50].

A wide variety of integration intervention strategies were used the term ‘integration. These included integration initiatives on governance, policy and legislative reforms, strategies for coordination of care among service organizations and networks, multi-disciplinary teams, use of new or existing cadres of staff for care coordination in facilities, among service organizations and in communities, revised roles and responsibilities for team members, task-shifting and task-sharing with non-specialist health providers, integrated treatment guidelines, training, supervision and mentorship for staff, infrastructure support (e.g. for co-location of comprehensive PHC services) and funding for integrating services and providing incentives.

We present the findings under three main themes. In Table 3 we provide an overarching finding for each of the three themes, and we provide an overall assessment of confidence for each finding. All findings had a similar rating of moderate confidence level, meaning that, based on the studies we reviewed, it is likely that each of the three findings is a reasonable representation of the underlying evidence contributing to that finding. In Additional file 5, we detail the application of the four GRADE-CERQual domains to show how we arrived at this assessment. In sum, we downgraded findings from high to moderate confidence for reasons related to minor to moderate concerns in areas of methodological limitations, relevance, and adequacy. Minor methodological limitation concerns were based on the CASP ratings (only one study had moderate to severe quality concerns and most studies had no to minor concerns). The main reasons were limited depth of qualitative data [35, 38, 40, 48]. Minor concerns about relevance related to the heterogeneity of intervention types with fewer studies focused explicitly in NCD-CD integration. Minor to moderate adequacy concerns were due to depth of data and the granularity of sub-findings (with studies contributing to different components of the synthesized finding).

### Outline of findings

The findings are categorized along three main themes: A. Policy alignment and Governance; B: Health systems readiness, intervention compatibility and leadership, and C: Human resource management, development, and support.

With respect to the applicability of the findings to integration of non-communicable, with long-term communicable disease (CD), there are three intersecting sets of implementation considerations. First, the broad findings on policy and governance are generic to any major health reform, including the importance an enabling policy environment, and a policy environment that is aligned in support of implementation across the health system. Second, given how complex it is to implement integration of NCD-related services in a PHC setting, important findings are those related to alignment of health system readiness, the fit of the new intervention with the current organization of services, and the key role of transformative leadership. Longitudinal care for NCD-related integrated services can be complex to deliver and attention should be paid to the findings on human resource management, development, and support.

#### A: Policy alignment and governance

Appropriate governance (political, legal, and administrative), that includes an enabling policy environment and policy alignment across management levels, as well as alignment with health system support services, are key to enable implementation and for sustainable integration reforms [14, 32–48].

##### Governance and an enabling policy environment

A clear governance framework is needed for integration reform, to provide it with a political, legal, and administrative foundation. Ideally the framework should be aligned across political, policy and implementation stakeholders, and aimed at strengthening multisectoral linkages between planning and monitoring and evaluation of services [38, 44].

“Governance need policies to strengthen multi-sectorial linkages in the planning, implementation and monitoring and evaluation of services.” [36](p5).

A favourable policy and implementation environment can increase the chances of successful and sustained integration reform. Meaningful collaboration and clear communication throughout implementation is required to promote ownership and narrow the gap between policy makers and implementers [34, 38].

“It is important to close the gap between policy makers, managers, providers and users through the generation of interactive spaces for dialogue and for the exchange of ideas and solutions." [38](p6-7).

An enabling policy environment is one where policy support makes integration reform more politically and practically feasible (including suitable adaptation to local context), which can help to narrow the policy to implementation gap [32, 36, 38, 43]. This includes having a policy environment is where there is shared expectations, staff communication and readiness to be support integration efforts, as explained:

“the extent to which there is a shared perception that integrated care is expected, supported and rewarded within an organization; communication between providers or staff within an organization to support integrated care; and variation in workforce readiness to implement models of integrated care.” [34](p8).

Part of an enabling policy environment is a supportive and stable policy climate, without frequent disruptive changes. A stable policy environment may increase the confidence and commitment of stakeholders and the likelihood of success, while frequent policy changes can negatively affect stakeholder commitment [36, 38], as noted:

“Frequent policy reversal can threaten the progress and commitment of network members.” [38](p6).

By way of example, it was reported that frequent policy changes in South Africa made it harder for stakeholders to buy into the PRIME project on integration of mental health services into primary health care [36]. Having a gradual approach and logical sequencing of implementation may also better motivate staff engagement, giving them time to trust the integration initiative [38].

It was noted that while having a supportive policy framework is important for guiding integration programmes, this should not limit initiation of integration efforts [38]. Integration efforts can also start as informal agreements among local stakeholders, which may then evolve into more formal arrangements with the necessary policy and political backing [38].

Though no interventions included patient and community participation, several studies referred to the value of patient and community participation, as a mechanism for accountability for the services, as well as an opportunity for patients to taken on self-care elements of their health [36–38, 47, 48], as noted:

“Encouraging citizen and community participation through health education, self- care, social control, and satisfaction surveys is an important aspect of integration strategies.” [38](p6-7).

##### Alignment with the health system

Implementation of integrated service delivery also requires alignment of health system functions in support of implementation, such as finance systems, human resources systems, infrastructure, and supply systems and information systems [4, 20, 21, 23–25, 29, 33, 34, 36, 49, 51].

**Financial systems**

Regulations, funding, resources, and payment mechanisms should be aligned to support implementation and improved quality of integration services; poor alignment has been implicated in impeding of integration efforts across several country settings [33, 35, 36, 43, 44]. Funds are required for structural, or ‘hardware’ resources (infrastructure, human resources, information systems, and medicine and equipment), as well as for ‘software’ resources (for leadership, supervision, and building multi-disciplinary teams) [33, 38, 41, 46]. Funding and payment mechanisms (such as health insurance schemes) should also incentivize delivery and use of integrated care [33, 41]. For example, financial schemes should provide adequate reimbursement for integrated care to incentivize providers and patients [41], and community health workers should be adequately compensated for taking on dual roles [33].

Government funding might have to be increased over and above donor funding to sustain integration [14, 32, 38]. Further, government and donor funding would need to be harmonized and aligned with national plans, and it should be guided by principles of effective international funding [38]. This will help prevent further fragmentation and unequal quality in health service delivery, especially in countries dependent on donor funding [14, 32, 38]:

“… it is crucial to harmonize and align donor funding around national health plans and strategies following the principles of the Paris Declaration on Aid Effectiveness: ownership, harmonization, alignment, results, and accountability.” [38](p9).

**Infrastructure**

In LMIC settings, lack of facility space for consultations may pose a challenge for delivery of integrated services, as there is sometimes insufficient space for clinical consultations [47, 48]. While formal consultation spaces and formal team collaborations are important, spaces for informal engagement between multiple providers are also helpful for promoting collaborative practices. This was illustrated when primary care organizations were co-located in a ‘super clinic’ in Australia, that was purpose-built integrated primary care delivery [46]. Nevertheless, health workers found that opportunities for social and chance encounters among different service organizations were still limited; and it was these informal encounters that they valued as promoting collaborative practices amongst staff across different organizations [46].

**Supply systems**

Integration of different health service programmes can lead to a need for increased medicines and supplies, such as diagnostic test kits for health screening, or medicines for a newly integrated health service [14, 36, 38, 48]. For example, additional supplies for chronic medicines for NCD health care, or new psychotropic medications for new mental health services may be required to support integrated service delivery. Strong procurement systems are needed to ensure adequate provision of supplies to support the integrated delivery of services [14, 36, 38].

**Health information and monitoring systems**

Integration initiatives need strong information systems to facilitate safe and efficient exchange of shared patient and service information for collaborative clinical care, as well as for monitoring and evaluation of implementation progress and outcomes of integrated services [14, 34–36, 38–41, 43–47].

Being able to access patient information in one place is highly valued for how it can support delivery of integrated care. Information communication technology (ICT) can play a role in facilitating shared access of patient and service records within and across service systems and institutions, through, for example, use of electronic health record systems (EHRS), telemonitoring systems, and web-based resources and tools [34, 36, 39, 40, 44, 46]. It was reported that integration of services in the Pacific Islands benefitted from having an electronic information system:

“Use of public domain software…enabled team members to establish diabetes registries, input data, and generate clinical reports to monitor trends (individual and aggregate), guide management decisions, and develop strategies.” [40](p7).

However, ICT can also impede service delivery, due to problems in information access, differential access by various professional groups, limited functionality of the available technology (e.g., interoperability, usability), and organizational and provider inertia to adopt and adapt to using ICT [38–40, 46]. Also, staff may be concerned about compromising patient confidentiality and privacy, especially in mental health services [39], especially if it requires sharing records across organisations [46]. Continuous staff training may be required for balancing effective, efficient collaborative care with protections for patient privacy. This may require organizational agreements between health care services to ensure that information exchange is well regulated [39, 46, 47]. More training may also be needed to enable all providers to use electronic information systems for managing and monitoring work [38–40].

Monitoring systems are needed to track the implementation progress of integration and to track standard measures of patient and population health outcomes, including referral and follow-up care within and among health service delivery platforms (e.g. hospital, primary and community care) [14, 34–36, 39, 40, 43, 44, 46]. However, requirements for documenting integrated care may also become overwhelming for providers, for example if multiple forms are asked for and large amounts of data is requested. Complex documentation demands may become more challenging in the absence of functional shared electronic information system [40, 47, 49].

Nevertheless, health informational support is key for implementation of integration programmes, and it is needed along the full continuum from planning the intervention, to monitoring, to evaluation of progress. An example from a polypharmacy management project illustrated how pharmacists first gathered information to understand the extent of the problems related to clinician prescription patterns to make them aware of the problem. They also used data systems to monitor the outcomes of the polypharmacy management intervention, including using data to illustrate early gains and encourage staff [44]:

“ It is important to demonstrate early gains with measurable results and pilot experiences to encourage and sustain efforts to move forward with the integration process.” [38](p6).

It was noted that monitoring system should aim to be service user-centred, by for instance, including measurements such as patient satisfaction with integrated care [34, 38].

#### B: Health systems readiness, intervention compatibility, and leadership

Health system readiness for change, compatibility of the new integration intervention with existing organisational practices, and the quality of leadership are key factors that can influence the success of integration [14, 32–50]**.**

##### Assessment of health system readiness for change

Health system functioning and readiness influences success of integration and should be assessed prior to implementing new initiatives. Stronger health systems may be better able to integrate interventions [38].

“Integration efforts seem to be more successful when implemented in the context of stronger health systems with less structural problems (e.g., systems with a strong steering role of the national health authority, less segmentation, and adequate levels of financing)." [38] (p6).

Health system readiness should include assessment of structural resources (hardware), as well as the organizational culture and relational resources (software). Assessment should also include identifying barriers and facilitators to supply side issues (such as organization and accessibility of health services), as well as demand side issues (such as patient awareness of service options, patient health literacy levels, perception of quality of the services; accessibility in terms of transport and costs; use of alternative and traditional health services) [32, 34, 38, 39, 43, 44]. Health system strengthening may need to be prioritized in countries with weak health systems and large donor funded presence, as donor-associated vertical programs may result in fragmented and uneven health system performance [38].

##### Intervention compatibility and ‘goodness of fit’

Several case studies emphasized the importance of high compatibility, acceptability, and feasibility of integration, also referred to as ‘goodness of fit’ [32–37, 39–50]. Goodness of fit is described as the degree of fit between the characteristics of the integrated care innovation, and the extent to which it is compatible with the organisational practice and environment (in terms of patient care processes and preferences of staff and patients) [34].

Several contextual elements in health systems and the implementation environment influence the fit of the new integration intervention with existing practice. Analysis of a number of case studies lead Stadnick and colleagues [34] to identify the following common elements: the extent of siloed or verticalized services before integration, whether clinicians have a narrow clinic role and identity versus a broad ‘whole patient’ view, stakeholder agreement on prioritizing integrated care and patient groups, and whether their support was aspirational and discordant with their actual practices, the extent of collaborative communication amongst leaders and service stakeholders, and the extent and nature of patient advocacy and community involvement at all stages of integration [34].

A range of barriers and facilitators of goodness of fit has been identified across several, diverse integration intervention studies. Barriers relate to poor management of shifts in roles and responsibilities inter-professional tensions, staff shortages, poor patient-provider relationships [36, 41, 49], staff shortages and insufficient training [35, 36]. For instance, in one study, a multi-disciplinary team could not be employed to train others to integrate mental health into TB and maternal health services in South Africa [35]. In China, lack of patient awareness about the new intervention to introduce cardiovascular services into diabetes care, reportedly lead some patients to became suspicious that doctors may have a financial incentive for prescribing cardiovascular preventive medicines [41].

Facilitators of goodness of fit include factors such as good quality and flexible training and specialist support [36, 45], availability of integrated clinical guidelines that simplified comprehensive care delivery, staff satisfaction in providing comprehensive care that they feel better meet patient needs, and staff’s sense that integrated services increased clinical efficiency and effectiveness [33, 36, 40, 45]. In South Africa, doctors and pharmacists who implemented new medical adherence clubs for integration HIV and NCD medication, differed in their appreciation of the intervention; with clinicians feeling it freed them up to concentrate on specialized clinical support, while pharmacists felt concerned about their increased workload with prepackaging medications [33].

##### Transformational leadership

Integration is a complex health system reform and requires leadership to drive the transformation required, and that supports a shared vision of participation, collective action, and teamwork amongst staff delivering the integrated care [34, 36, 38, 40, 42–46].

Given the complexity of change required for integrated delivery of services, an organizational culture that is “outward looking” or open to innovation is essential [44]. In an intervention to integrate prevention of cardiovascular disease with diabetes care in China, an external partner commented on the complexity:

“Scaling up primary care … is a major challenge … requires careful planning … matching workload to resources, extending beyond diabetes [only]… and showing impact on population health.” [40](p20).

Transformational leadership allows for balancing a centralized, structured and directive leadership approach at the macro level (country and systems) and meso level (across institutions), with allowing staff to exercise more flexibility at the microlevel of clinical leadership and frontline service delivery [34, 36, 38, 42, 43]. Such transformational leadership can promote local adaptation and innovation. For instance, while a hierarchical management and a strict medical culture can promote standardization and control of integrated service delivery, it may also lack the flexibility that staff need to solve difficulties at the front line [36, 43]. There may sometimes be tensions between these different approaches:

“I think there will always be tensions between the people who are in commissioning and managing roles who want to manage the process to get the results delivered as quickly as you can, and the people who are clinicians who are delivering it trying to get the balance, realistic time scale and not letting the thing wallow and not deliver. But I think it is a constructive tension. [43](p135).

Transformative leadership is supported when there is shared leadership that is aligned across levels, and where there is strategic planning and knowledge of change management at all the management levels [34, 43]. Change management skills and strategies that have been identified as helpful include: setting realistic expectations, managing expectations, ensure all leaders have project management support, engagement with providers focused on supporting them, regular communication with and constructive feedback to providers, and demonstrating early gains to encourage and sustain integration, mechanisms for monitoring and evaluation [36, 38, 40, 43, 45, 46].

Further, transformative leadership can provide clarity on team and individual staff roles and responsibilities, build meaningful and sustained partnership across organizations, and develop inter-disciplinary teams and collaborative care practices [43] The role of senior leadership, together with the transformation team, would be to make the business case for integration, secure the resources required for implementation, and promote collaboration across different levels of health service delivery [43]. Facility managers are key stakeholders for championing the integration initiative, to influence people at the frontline and to drive the operational management of changes. Clinician-led leadership and champions are considered particularly useful for credibility of the intervention and buy-in amongst clinical staff [40, 43, 44].

#### C: Human resource management, development and support

Human resource management, development and support are major considerations for implementing integration of services; with the need for strategic realignment of financial, management, human resource systems to support the integration reform objectives. This involves human resource availability, appropriate skills and staff training, as well as supportive management and supervision [14, 32–37, 39–41, 44, 45, 47–50].

##### Human resource availability

Integration reforms require availability of appropriate human resources, both in terms of adequacy of numbers, but also in terms of configurations of leadership and of co-ordination, staff skills mix and staff distribution [14, 32–36, 44, 47–49]. Shortages of human resources are often due to limited funding, but it is also due to staff rotations, shifts, preferences, absenteeism, staff skill mixes and shortages of skills [33, 35, 36]. In terms of staff availability and distribution, there are indications from integrating HIV and reproductive health services case studies that client to provider workloads were better matched in better performing facilities [32]. Nevertheless, these case studies also indicated that other factors beyond patient load were relevant, such the quality of leadership and management support. It was noted that these management factors were important for success, even when resources were limited [32]:

“… managing stock-out, staffing distribution, rotation of staff and the way external support is provided (supportive supervision and vertical donor support) affect ability to provide integrated care.” [32](p13).

Organization of staffing, such as changing employees’ roles and responsibilities to facilitate integration, requires careful management, as it is associated not only with the skills of providers but also their attitudes to their changing roles and functions [32, 34, 47, 49]. For example, when new cadres of workers, such as community health workers were introduced as part of integrated services, alignment of new and current staff roles became challenging [47, 49].

##### Staff attitude, skills, and training

The individual and organizational characteristics of service providers may determine their ability to provide integrated care [34]. Provider characteristics that shape implementation include provider knowledge, skills, and professional training in integrated care, as well as their level of confidence in, and motivation for providing integrated care. Clarifying provider roles and providing good quality training can help to strengthen those provider characteristics [14, 33–37, 39–41, 45, 47–50]. In settings where there may be limited services and resources for managing NCDs, it may be more challenging for health workers to build confidence in providing integrated NCD services [35, 37]. Reasons include limited skills in newly integrated disease programmes and uncertainty about the value of integrating service delivery. Similar challenges were noted in a programme to implement delivery of integrated NCD services in Malawi [37], and with integrating mental health with TB and maternal-child services in South Africa [35]. Further concerns were poor literacy about mental health among PHC staff, as well as concerns about stigma when integrating mental health care with TB care in South Africa [35].

Good quality training and capacity development of staff are important considerations for supporting integration initiatives, as noted earlier. Good quality training has been characterized as needing to be comprehensive, tailored, flexible and interactive [40, 45]. It should include clinical skills training for newly integrated services, as well as mentorship and specialist support. Tools such as integrated clinical guidelines to promote collaborative practice [36, 40] can be helpful. Skills in techno-literacy can also help staff to effectively use shared information and electronic communication technologies for managing and monitoring their work [39, 45, 47]. Skills building should include training on how to educate and communicate with patient communities about the value of the newly integrated services [33, 41].

Good-quality, diverse training by content experts was considered a good motivator for staff in supporting their implementation of integrated services [40, 41, 45]:

“Involvement of content-experts (familiar with NCD prevention and management in low-resources settings) helps strengthen and accelerate consensus development and adoption of evidence-based standards of care and best practices.” [40](p7).

However, a shortage of specialists to train others in a multidisciplinary team can limit the development and thus the support of front-line staff [35, 36]. For instance, integration of HIV and TB care services for pregnant women in South Africa was limited by inadequate specialist clinical training for nurses, which interfered with nurse-initiated management of anti-retroviral treatment (ART) [48].

##### Human resource support and supervision

Successful and sustainable integration requires careful engagement with stakeholders throughout integration reform process; it depends on personal, social, and organizational factors, including the quality of interpersonal and inter-institutional relationships [32, 36, 38–40, 42–44, 46]. The quality of interpersonal relationships and relationships between collaborating institutions can influence willingness of stakeholders to participate in the integration initiative:

“The success of integration efforts is strongly associated with the quality of interpersonal and inter-institutional relations, including a commitment and willingness to change; a sense of belonging and appreciation; trust and communication; and credibility.” [38](p6-7).

Supportive supervision and mentoring are considered key elements for engaging and motivating providers. It was noted that supervision that take only a technicist ‘tick-box’ approach (such as, for example checking supplies and guideline use only), will have limited results. It is recommended that supervision should rather be aimed at developing staff skills and experience to enable them to make good decisions about patient care [32]. This is illustrated in case studies on integration of mental health in a few LMICs, where supervisors took a more “paper-based”, tick-box approach to supervision, which left staff feeling overburdened and unsupported, while in another setting, good and regular communication and coordination on the part of the supervisor, left staff feeling empowered to solve problems at the frontline [24].

Effective provider engagement and support for implementing integration would require support for both structural (‘hardware’) resources as well as organisational culture and relational resources ‘(software’) [32, 36, 42]. System ‘hardware’ may include financial, infrastructure, supplies and technical resources needed for a functioning health service. System ‘software’ includes processes for development and maintenance of collegial, collaborative, multi-disciplinary teamwork, needed for delivery of integrated services. Such collaboration requires trust amongst frontline colleagues, and between frontline workers and management [32]. A study evaluating reasons for better performing integration implementation notes that facilities with limited ‘hardware’ but good ‘software’ resources (such as good leadership and teamwork), may outperform facilities with sufficient hardware resources, but where there is poor leadership [32]:

" In other words, the systems software (people) can affect how effectively the systems hardware is utilized to deliver integrated services.” [32](p12).

The common “software” elements that were found in better-performing facilities include strong, supportive supervision, regular rotation of staff among services and motivated, confident staff who can work in teams [32]. Commenting on the influence of relational ‘software’ elements (such as trust) on implementation, the author noted:

"In our study, the relationship of trust between colleagues appears to be at least as important as that between frontline workers and managers.” [32](p13).

Integrated service delivery often require collaboration between teams from different disease programmes or working across disciplinary areas care [36, 40, 44, 46]. For example, in a polypharmacy management intervention, doctors and pharmacists needed to work together on integrated medication review services [44], and in others, clinical staff collaborated to provide seamless screening, treatment and or referral services for mental health at primary care level [36]. Collaborative teamwork sometimes involve tensions between professional groups, with each group questioning the other’s competence or having concern about their roles being usurped [44]. Commenting on challenges of multidisciplinary teamwork in the polypharmacy management intervention, one clinician commented:

“Teamwork is not easy: we need to find a trade-off between hierarchy and teamwork.” [44](p12).

Managing professional tension requires focused investment in team building for inter-professional practice and developing a shared vision [44, 46]. It was noted that even when there is a shared vision, teamwork may require active guidance to promote teamwork [44], or even a memorandum of understanding on working collaboratively [46].

## Discussion

The aim of this rapid review was to synthesize qualitative evidence on implementation considerations integration of NCD-related services into primary health care services, from the perspective of health workers, managers, and policy makers. The evidence covered a wide range of countries, from both high and low and middle-income settings, and from a variety of disease programmes and implementation strategies for integration.

The findings highlight the important role of and enabling policy environment where governance mechanisms and policy are in support of the integration reform. Policy alignment with health system support services such as financial, supply chain and health information systems provide further support for the operational elements of implementation. The importance of health system readiness for implementing a new and complex intervention was highlighted, including how the baseline functional level of the health system can determine the capacity and success of implementation. A related factor is the compatibility of the new integration reform with the existing health service organisation, and the need for a ‘good fit’ with routine health service delivery. Transformative leadership is highlighted as important for leading, co-ordinating, and supporting implementation, and for change management, including managing both the technical (hardware), and the relational aspects (software resources) required for effective implementation. Finally, the review highlights the crucial role of effective managing, development, and support of human resources. This includes not only adequate staff availability and appropriate staff skill mix, but also development of staff knowledge and skills to ensure capacity, confidence, and motivation for integration, and the need for supportive supervision. Studies acknowledged the importance of community and patient involvement in planning, implementing, and monitoring integration, though interventions did not include such patient involvement.

When considering the applicability of the findings to LMICs in particular, key considerations are high-level political and leadership commitment, policy alignment with resources, harmonization of government and donor funding to prevent further fragmentation, and alignment with national plans (also to prevent further fragmentation), and information and monitoring systems. A review of primary care models for NCD interventions in Sub-Saharan Africa largely concurs with our review findings, especially regarding the importance of training, multidisciplinary teams, and supportive supervision [52], but it also noted the risks of health providers being further overburdened [52]. A recent (2021) review on integration models for diabetes and hypertension in LMICs showed the uncertain effects and noted the importance of programmes considering context-specific factors related to health systems and populations [9]; and our review contributes health systems insights.

Our main themes were roughly clustered according to the WHO health system building blocks (for example governance, leadership, human resources) [31], and covered several of the key categories in the SURE framework (such as provider knowledge, skills and attitudes, leadership, and health system constraints) [30]. A scoping review of models and programmes of integrated care for multi-morbidity found a similar clustering of implementation considerations around the WHO health systems building blocks [26]. The implementation considerations identified from the perspective of providers in this review largely align with strategies identified in synthesise of other implementation frameworks (such as policy and leadership, financing, alignment with the health system, goodness of fit, capacity development, provider engagement) [21, 22], as well as with the strategies in the framework on integrated, people-centred health services (IPCHS framework) developed by the World Health Assembly in 2016 [53].

The findings in our review are also in alignment with findings from several reviews on different aspects of integration interventions [54–57]. In a review of governance models for integration of PHC and secondary care services in high income settings, Nicholson and colleagues identified ten elements of health care governance, much of it overlapping with findings from our review, such as alignment of vision, planning, resources between leaders, managers and local health providers, as well as need for skills building and collegial collaboration [54]. A review on continuity of chronic care for in remote rural Australia highlighted similar resource issues, and stressed the importance of skilled staff which local cultural knowledge [55]. In a review of experiences of interprofessional team members caring for older adults, the authors note as we did in our review, the importance of the relational elements of building a multi-disciplinary team and change management [56]. Their review provides further insights on the inevitable stressors, tensions, territoriality, and unequal power dynamics associated with interprofessional team dynamics. These include, for example, the shift in thinking and practice required when combining teams; valuing others’ and one’s own contribution to the team, learning to communicate by developing a shared vocabulary, communication that is courteous, respectful, and able to “challenge one another.” [56]. The review notes that team approaches can be strengthened by “appreciating the life world” of the patient and on all staff focusing core business on meeting complex patient needs that can improve patient quality of life [56].

### Implications for future research

Integration is a complex health reform, and the experience of stakeholders is important for understanding the barriers and facilitators. We observed a wide variety of integration interventions in terms of different configurations of disease programmes (e.g., hypertension and HIV services), as well the intervention implementation strategies (e.g., introduction of screening services, multi-disciplinary team collaboration, new cadres of health workers, task-shifting etc.,). This heterogeneity of integration interventions has been found in other reviews of PHC integration models [8, 14, 52, 58]. The heterogeneity, as well the variation in the level of intervention description, makes it harder to compare findings across interventions. Given the complexity of integration interventions, implementation research and practice would benefit from more conceptual clarity supported by theoretical frameworks, as well as rigorous study methods and reliable outcome measures [9, 14, 59]. Future studies can benefit from ongoing efforts to refine a taxonomy of implementation strategies to promote conceptual clarity, relevance, and comprehensiveness of implementation strategies, for use in research and practice [21, 22].

The number of eligible studies identified for this review indicates that the evidence base is growing. While cross-sectional stand-alone qualitative studies of provider experience provide an important perspective, given the complexity and long-term nature of integration reform, we need more innovative, in-depth, longitudinal studies, using for example, ethnographic method and theoretical frameworks. More detailed descriptions of the intervention components and the local health care context is required (perhaps drawing on a taxonomy of implementation strategies), to allow for appraisal of the applicability of findings to similar settings. In a recent effectiveness review of integrated services for diabetes and hypertension in LMICs the authors noted the challenge of heterogeneity of interventions and called for more rigorous evaluations that include implementation research, economic evaluation and qualitative research on barriers and facilitators [9]. Finally, further implementation research is also needed, on how to involve patient and communities including local governments during planning, implementation, monitoring, and reviews point to integration services enhancing patient access to care and patient satisfaction [60].

### The strengths and limitations of the review

Rapid reviews are valued for providing health decision-makers with timely access to evidence, though streamlining systematic review methods make it susceptible to bias [23]. Our review was embedded in a larger, ongoing systematic review, with a published protocol that was adapted for this review. Embedding the review had pragmatic benefits as it allowed us to quickly have access to a large data base of records, a set of inclusion and exclusion criteria, and an initial set of records that had already been reviewed by two reviewers (double screened). As shown in description of our methods in Table 1, we also built in quality assurance measures, such as second reviewer checks. Drawing on a pre-existing set of search records and other deviations from the standard rapid review processes were due to pragmatic and logistical reasons of feasibility and time, and it also has limitations. Other deviations are: creating a smaller, more manageable sampling frame before final sampling, sourcing additional records from experts, and doing formal quality appraisal only the studies selected for extraction, which could have skewed the sample. Embedding the rapid review in a larger review include the following limitations. First, selection of articles for the rapid review was initiated before the initial screening for the parent review was completed, meaning that several hundred additional articles that may have been relevant for the rapid review were never screened. Second, part of the screening for titles that was undertaken (the ‘double screening’) was aimed at evaluating eligibility for the parent review and not specifically for the rapid review and it is possible that some articles relevant to the rapid review were missed during that process. A related limitation is that that the search terms to identify NCD-related interventions within the parent review database may not have been an exact fit, so studies could have been missed. Selection of the final sample was done from the studies in a sampling frame (*N* = 51), which was a sub-sample of the total number of eligible studies (*N* = 81), and the sample size was small which are further limitations. Drawing on additional WHO expert resources is not standards and could have skewed the sample. Finally, while our findings are based on wide scope of integration studies, we were not able to update our search to account for studies related to integration of services that may have been implemented address health service related to the COVID-19 pandemic. There are likely important recent findings with COVID pandemic-specific implications that are no captured in this search, and this calls for a further review of Covid-related service integration.

Nevertheless, the studies identified covered a large, diverse geographical spread, income settings, different configurations of disease programmes being integrated and a variety of integration implementation strategies and appear to reflect both the homogeneity and similar implementation considerations noted in other reviews [9, 21, 26, 52, 54–56]. We received feedback from WHO working group on the draft findings which was an opportunity to check the relevance and applicability of the findings. Our rapid assessment of the confidence level of findings provided decision-makers with additional guidance on the utility of the evidence.

## Conclusion

The rapid review produced qualitative evidence on factors influencing implementation of NCD-related integration into PHC, across diverse country settings and integration intervention types. The review findings presents insights on how health workers responses may be shaped by the complex interaction of individual, social, and organizational factors that may be specific to the context of the intervention and show the importance of cross-cutting issues such as policy alignment, supportive leadership and health system constraints. This knowledge on the dynamics influencing health worker responses can inform the development of future implementation strategies and implementation research.

## Supplementary Information

**Additional file 1: Supplementary file 1.** NCD-related PHC Integration review: PRISMA Checklist.

**Additional file 2:** A: Medline search strategy used for the Cochrane (parent) review on Health care worker perceptions and experiences of PHC integration [15]

**Additional file 3:** Criteria for inclusion and exclusion of studies for screening.

**Additional file 4: Additional file 4.** Characteristics of papers included in the sampling frame.

**Additional file 5:** Table:CERQual assessments for seven main findings of the PHC Integration rapid review.

## Data Availability

The datasets used and/or analysed during the current study are available from the corresponding author on reasonable request.

## Abbreviations

CASP: Critical Appraisal Skills Programme
CERQual (or GRADE CERQual): Confidence in the Evidence of Reviews of Qualitative Research
CDs: Communicable diseases
LMICs: Low-and middle-income countries
NCDs: Non-communicable diseases
PHC: Primary health care
SURE: Supporting the Use of Research Evidence
WHO: World Health Organization
