# Supplementary material for: Implementation considerations for non-communicable disease-related integration in primary health care: a rapid review of qualitative evidence
Source: BMC Health Serv Res. 2023 Feb 18;23:169. doi: 10.1186/s12913-023-09151-x (PMC9938355; doi:10.1186/s12913-023-09151-x)
Supplement: Supplementary file 2 — Additional file 2: A: Medline search strategy used for the Cochrane (parent) review on Health care worker perceptions and experiences of PHC integration [15] [file 12913_2023_9151_MOESM2_ESM.docx]

**Supplementary file 2**

## **A: Medline search strategy used for the Cochrane (parent) review on Health care worker perceptions and experiences of PHC integration [15]**
