# Supplementary material for: Implementation considerations for non-communicable disease-related integration in primary health care: a rapid review of qualitative evidence
Source: BMC Health Serv Res. 2023 Feb 18;23:169. doi: 10.1186/s12913-023-09151-x (PMC9938355; doi:10.1186/s12913-023-09151-x)
Supplement: Supplementary file 3 — Additional file 3: Criteria for inclusion and exclusion of studies for screening. [file 12913_2023_9151_MOESM3_ESM.docx]

**Supplementary file 3**

**Criteria for inclusion and exclusion of studies for screening**

(Adapted from Moloi et. al., PHC Integration, Cochrane parent review, in progress)

**Healthcare workers’ perceptions and experience of factors influencing implementation of PHC integration interventions, with a focus on integration of NCDs with communicable disease (HIV/TB), and with sexual and reproductive, and maternal health: Rapid systematic review of qualitative evidence.**

**Version 1.0 for NCD_PHC Integration review: updated 28 July 2020.**

**Criteria for considering INCLUSION of studies for this synthesis.**

| **Study design/methods of studies**   - **Primary studies that use qualitative study designs**   (Such as case studies, grounded theory studies and qualitative process evaluations)   - Studies that use **qualitative methods for data collection**   (For example, focus group discussions, individual interviews, observation, diaries, document analysis, open-ended survey questions-where qualitative data is analyzed qualitatively)   - Studies that use **qualitative methods for data analysis**   (For example, thematic analysis, grounded theory).   - Mixed methods studies (e.g., effectiveness studies with a qualitative process evaluation and or use of open-ended survey questions- where it is **possible to extract the qualitative data that were collected and analyzed using qualitative methods.** - We will **not exclude any studies based on our assessment of methodological limitations** but will **utilize this information to assess our confidence in the synthesis findings**.   **PHC-parent review Team decision: 15/04**  When we have a study that is based on the experience of one HCW, as narrated by that HCW himself/herself, this may be considered as primary data. We would have to check the Full-text to see the details and to make the final determination (e.g., whether the person is writing an opinion piece (Excluded), or using a self/auto-ethnographic method (Included) |
| --- |
| **Phenomena/Topic of interest**   - Studies that report on **the perceptions and experiences of healthcare workers who were/are involved with implementing PHC integration (as active participants in implementation or as recipients/end users)** - **And/or** **perceptions and experiences of management and policy makers who are making policy and or in a management/leadership or supervisory role in the PHC integration interventions.** - Furthermore**,** we will also extract **perceived solutions or ideas to these barriers as per healthcare worker suggestions.** |
| **Participants/population**   - **Healthcare professional workers** on both **healthcare provision and management levels** (frontline clinical workers and supervisors, managers, policy makers) - **Lay health workers** (as defined by Lewin et al 2010.) Definition of lay health worker: “Any health worker carrying out functions related to healthcare delivery, trained in some way in the context of the intervention, and having no formal professional or paraprofessional certificate or tertiary education degree.” (Lewin et al, 2010). - **Any other individuals involved in supporting the provision and management of PHC integration interventions**. These individuals could include administrative, managerial, and supervisory staff. - **Non-specialist/generalist clinical health professionals** delivering services at a PHC site.   **Decision: 16 July 2020 (with Hongyi Xu)**   - Specialist health care staff are INCLUDED, **only if they are involved with delivering the PHC Integration service in some way. And or, they are involved with an intervention where there is a “whole-health system” intervention, that is focused on improving the linkages and co-ordination between PHC and hospital services, And the focus is on improved PHC delivery.** - For example, where specialist health care staff (like a cancer or cardiac specialist doing outreach to PHC) are providing first line primary care, INCLUDE for full text review, and then check if this is considered a primary care service in that country context. (For example, routine child immunizations service provided by a pediatrician). * (See Criteria for EXCLUSION below) - Or, where **Hospital and PHC management and staff work together to improve continuity of care between hospital and PHC care,** but where the focus of the Integration intervention is improved care at the PHC level. |
| **Intervention of interest**   - We define integration as   **Organizational changes and modifications of health system functions to create connectivity, linkage, alignment, and collaboration in the delivery of one or more health service.**  Our definition expands on and remains compatible with the definition used in the Cochrane effectiveness review (Dudley 2011). Dudley and Garner (2011) defines integration as  “***a way of delivering a series of targeted technologies and interventions together that sometimes have been delivered as a series of “vertical’ programs***” [9]   - **The integration could allow clients to receive multiple services during a single visit, either from a single health worker or different health workers.** - **Integration may allow the extension of a package of care services, where this was previously limited.** For example, providing TB testing for all HIV patients, or chronic disease screening for all PHC patients, irrespective of their presenting complaint. These services do not necessarily need to be presented on a same day visit or by one HCW, but it should be considered a change in policy and practice/ a health reform around service delivery, that required integration of one or more health system functions (**see examples below).**   **FOCUS on NCD-related PHC integration**  The topic focus is on integrated service delivery of **full spectrum of PHC services:**   - P**romotion, prevention, curative, rehabilitative (& palliative- though less so),** - Of **various non-communicable diseases**, amongst itself, as well as with other infective diseases/disease programs (e.g., HIV/TB), and with **sexual and reproductive health services** (e.g., STI, **maternal health** services) - Where **other levels of the health care system are involved, the focus and outcome of the intervention should remain improved PHC service delivery** **(e.g., see Participants- team decision 17 July).**   **Approaches to PHC integration**  We are interest in **various approaches to PHC integration.** Examples of integration may include but not limited to:   - We are interest in **various approaches to PHC integration**. Examples of integration may include but not limited to: - Clients can **receive multiple services from different healthcare workers** but delivered during a single visit. This is through mechanisms such as referrals and coordination. - Clients may receive **multiple services from a single healthcare worker** during a single visit (World Health Organization 2016). - An innovation in service delivery requires workers and managers to **increase co-ordination and linkage of their services**, for example, **extension of scope of work of existing cadre** of HCW, or **introduction of new cadre of HCW.** - **New efforts to link previously separate and un-coordinated health services.**   (For example, services for managing TB, HIV and STI clients in an integrated and seamless service)   - Efforts **to expand routine care for certain client groups.**   (For example, routine TB screening for clients at their HIV care visits).   - **Expanding the range of integrated PHC services, by combining a new service with existing services.**   (For example, introducing, new mental health screening to routine treatment services).   - **Efforts to link aspects of routine care for certain client groups** (for example, offering existing HIV testing services to all TB clients or TB screening for all HIV positive patients) ·   **Degrees of PHC integration**   - We are also interested in **different degrees of implementation of PHC integration (whether full or partial).** (For example, HIV and Tuberculosis (TB) care is delivered and supervised by the same clinical staff in an integrated fashion, but the drug supply and finance systems may remain unintegrated, operating separately and in parallel). - And where the **different health systems building blocks may be fully or partially integrated** (for example integrated Service delivery and Governance/clinical leadership and supervision of NCDs and HIV services, but other Health System Building block may remain separate or partially integrated (e.g. Health information system, Drug and supply system, Finance systems)   **Parent PHC integration review Team decision 15/04:**  We are including **interventions where two types of medicine practices are offered by one type of HCW**. For example, where allopathic and alternative medicine is offered by general practitioners (refer to record Adams 2001)  **Decision: 28/04**   - PHC package of services: **We are using a HEALTH lens**. We are interested in the full primary care package of health services. We non-health services, aimed at addressing social determinants of health, are being integrated within a primary care service (e.g., social welfare, legal services, food security, housing security), **the focus of the outcomes should still be HEALTH**. - Rehabilitation, palliative services, curative care are services that can be offered at different levels of specialization. **For INCLUSION, these services would have to be offered at a primary care/first point of entry and or community-based level (and be delivered, primarily by non-specialist staff).** - Multi-sectoral integration: This is included if health is the focus and or outcome of the integration efforts between multiple sectors and or between multiple institutions. - **We will include studies on training**. If the training is in-service training as part of implementing the PHC integration intervention service, in integration. (Refer # 1399 - Aggarwal 2020) |
| **Settings**   - Integration that happens at **primary healthcare level**   We are guided by the definition of primary healthcare services as including **all therapeutic, preventive, promotive and rehabilitation (and palliative) services** **delivered at the first contact point of healthcare** (Awofeso 2004),   - **In any type of primary care setting**, including integration **in public setting, private healthcare settings, as well as in public-private partnerships**. - We will include PHC integration **in any country.**   **Decision 16 July with Hongyi Xu**  The focus will be PHC health service delivery at the **health facility/ clinic level (and not community-based, or workplace, or school-based, or care-homes for aged, addiction etc. and specialized rehab centers, step-down health facilities requiring specialist staff)**   - If not delivered directly at the PHC health facility site, **the delivery of the service should be directly linked to a PHC health facility/PHC district management initiative,** for example outreach services, community mobilization for awareness raising, or immunization campaigns. - Any country, however, we want to **ensure that LMIC country experiences are well represented in the evidence base.** |

**Criteria for considering EXCLUSION of studies.**

| **Type of studies**   - **Quantitative studies**. For example, **Effectiveness studies** using a range of quantitative research designs (including **Epidemiological and surveillance studies**) - Exclude studies that **collect data using qualitative methods, but do not analyze these data using qualitative analysis methods** (for example, open-ended survey questions where the response data are analyzed using descriptive statistics only). - Exclude studies that are **not based on primary collection and analysis of data** (e.g., Discussion document, Think piece, Commentary, Editorial) - **Systematic reviews, Literature review, Overview of evidence.** - **Where we find relevant SReviews:**  1. We will review the studies included and **include it for Screening in the Rapid review** 2. We will **review the findings of the systematic reviews**, **and use it to map, compare our findings from the rapid review** |
| --- |
| **Population**   - Exclude people who are **not health care professionals or lay workers.** - **Specialist health care staff** (not involved in delivering PHC care)   **Parent review team Decision after consultation with policy maker (Tracy Naledi) 28/04:**   - Exclude specialist health care staff providing specialist health care* - (* Where specialist health care staff are providing first line primary care, INCLUDE for full text review to determine if this is considered a primary care service in that country context) |
| **Intervention**   - **Studies that report on healthcare workers’ *anticipated e*xperience of PHC integration** (no intervention implemented, but only planned for; so, **Exclude, formative, intervention development, modeling work)** - Exclude **introduction of new ICT** (information communication technology)/ **Digital tools** **where that is the only component of the intervention. In other words, if the focus is only/ or mostly on testing a new ICT, and not primarily on integration of a service)**   **Decision with Hongyi 28 July 2020: Exclude also:**   - Interventions integrating health care with other types of care that does not have clinical health care at its focus, such as social care, housing, nutrition, legal services etc. - Interventions integrating behavior change intervention into PHC services. While this is often key to health promotion and prevention for NCDs, the scope is too wide for this rapid review. - Interventions not focused on primary health care facility as the site of the integration, such as integrated home care, integrated community care, and integrated care at residential care facilities and or step-down hospital and rehabilitation facilities. This includes palliative care services. - PHC integration focusing on integration of children’s health services. |
| **Setting**   - **Exclude if site of service delivery is not a PHC health facility/clinic/ or not related to the PHC service delivery outreach activities** (such as for example community mobilization for immunization). The primary service should be delivered at the health facility/ clinic level, and not community-based, or workplace, or school-based, or care-homes for aged, addiction etc. and specialized rehab centers, step-down health facilities requiring specialist staff). - If it is **not in primary health care setting**, e.g., hospitals and specialist hospitals, training institutions. - Exclude Academic/ professional **training institutions** |
