# Supplementary material for: Implementation considerations for non-communicable disease-related integration in primary health care: a rapid review of qualitative evidence
Source: BMC Health Serv Res. 2023 Feb 18;23:169. doi: 10.1186/s12913-023-09151-x (PMC9938355; doi:10.1186/s12913-023-09151-x)
Supplement: Supplementary file 4 — Additional file 4: Additional file 4. Characteristics of papers included in the sampling frame. [file 12913_2023_9151_MOESM4_ESM.docx]

**Additional file 4: Characteristics of papers included in the sampling frame**

| **Author, year (reference)** | **Country** | **Study design (qualitative data collected)** | **Study aim** |
| --- | --- | --- | --- |
| Cramm, 2013(1) | Netherlands | Mixed-methods design (Interviews) | “…we examined the processes and challenges of developing and implementing  cardiovascular disease management programmes  in the Netherlands.” (pg3) |
| Duner, 2011(2) | Sweden | Qualitative design (Interviews) | “This study aims to examine the process of implementing a new continuum of care model in a complex organisational context and illuminate some of the challenges involved.” (pg2) |
| Evans, 2018(3) | Canada | Qualitative design (interviews) | “The aims of this article are to identify where mindsets diverge among clinical and managerial stakeholders involved in the implementation of integrated care networks known as ‘Health Links’ (HLs) in Ontario, Canada, and to describe strategies to support stakeholders’ capacity to collectively learn and develop more convergent views.” (pg1) |
| Fitzpatrick, 2017(4) | Australia | Qualitative case study design (interviews) | “This research was undertaken to understand the dynamics of best practice integrated care for people with [severe and persistent mental illnesses] SPMI living in a small rural community in Australia.” (pg2) |
| Grudniewicz, 2017(5) | Canada | Qualitative design (interviews) | “Here, we examine the extent to which a complexity-compatible policy in the area of integrated health and social care stimulated new and productive interconnections, sensemaking, self- organization, emergence, and co-evolution.” (pg1-2) |
| Hayes, 2017(6) | USA | Qualitative design (Interviews) | The goal was to better understand how primary care providers approach – as professionals and in collaboration with their clinical colleagues – the risk assessment, treatment, and management of persons who are overweight or affected by obesity.” (pg3) |
| Henderson, 2018(7) | Australia | Qualitative design (Interviews) | This paper explores the impact of policy and funding models on capacity to deliver integrated care for older people with mental health problems in rural communities in South Australia.” (pg3) |
| Ingram, 2019(8) | Mexico | Qualitative design alongside a trial (Document review, participant observation, focus groups, interviews) | In this article, we describe lessons  learned from the evolving process of conducting a qualitative implementation study alongside a cluster RCT of Meta Salud Diabetes (MSD) (Diabetes Health Goal), a CVD prevention curriculum targeting uninsured patients with diabetes…” (pg2) |
| Jetelina, 2018(9) | USA | Mixed methods design (Observation and interviews) | We worked with practices and behavioral health (BH) clinicians to design a suite of electronic health record tools to address these needs (“BH e-Suite”). The purpose of this article is to examine whether implementation of the BH e-Suite changes process of care, intermediate clinical outcomes, and patient experiences, and whether its use is acceptable to practice members and BH clinicians.” (pg1) |
| Jones, 2017(10) | USA | Qualitative design (Focus groups) | “To describe HHC nurse perspectives about  challenges and solutions to coordinating care for recently discharged patients.” (pg 1). |
| Jones 2015(11) | USA | Qualitative design (Interviews) | “The purpose of this study was to understand  the challenges in coordination of care, as well as  potential solutions, from the perspective of hospitalists  and PCPs in North Carolina.: (pg1) |
| Kianfar, 2019(12) | USA | Qualitative design (Interviews) | “Our study objective is to identify and characterize care coordination activities performed for patients with [heart failure] HF and [chronic obstructive pulmonary disease] COPD.” (pg4) |
| Lalani, 2019(13) | UK | Qualitative design (Participant observation, interviews) | “This study aimed to examine the extent to which some of the principles of the Buurtzorg model could be adapted for community nursing in the UK.” (pg3) |
| Leppin, 2018(14) | US | Mixed method design (Participant observation, interviews) | “We sought to understand, categorize, and richly describe key challenges and opportunities related to integrating EBPs into routine primary care practice in the United States, using the CDSMP as a test case.”(pg2) |
| Limbani, 2019(15) | South Africa | Qualitative design alongside a trial (Observation and interviews( | “We report on a task shifting intervention using lay health workers to support clinic staff in providing chronic disease care in rural South Africa, where the HIV epidemic and an ageing population have increased demand for care” (pg1) |
| Lindeman, 2012(16) | Australia | Qualitative design (Observation, focus groups, interviews) | “This paper reports on specific findings of the evaluation relating to intersectoral collaboration  in Aboriginal communities, a key enabling factor of  effective dementia awareness and care.” (pg 2) |
| Lockhart,2019  (17) | Canada | Qualitative design (Interviews) | “…in this manuscript we qualitatively explore the dynamics of PCPs’ engagement in SCOPE, a voluntary care coordination initiative, designed to provide integrated care for patients with complex medical conditions.” (pg3) |
| Loeb, 2016(18) | USA | Qualitative design (Interviews) | “In order to better understand the day-to-day  challenges of PCPs, the strategies they use to meet these challenges, and how to better support PCPs, we sought to characterize [primary care physicians] PCPs experiences with complex patients.” (pg2) |
| Lowe, 2018(19) | Australia | Qualitative design (interviews) | “The aim of this qualitative research was to explore perceptions of organisational change related to the integration of nurse practitioners from key nursing  stakeholders.” (pg1) |
| Mayer, 2018(20) | UK | Qualitative design (Case note audit, interviews, observation) | “To describe the composition and processes of multidisciplinary care between maternity and cardiac  services before, during and after pregnancy for women with cardiac disease, and explore clinicians’ (cardiologists, obstetricians, nurses, midwives) and women’s experiences of delivering/receiving care within these models.” (pg1) |
| Murphy, 2018(21) | Vietnam | Qualitative design (Interviews) | : This study examines barriers and facilitators to the integration of services for depression in PHC from the  perspective of PHPs in one rural and one urban district  of Hanoi municipality, Vietnam by examining individual,  organizational and structural variables.” (pg2) |
| O’Conner, 2015(22) | USA | Qualitative design (Ethnographic participant observation) | “As part of an observational study evaluating the effectiveness and cost-effectiveness of an integrative,  team-based care model for the treatment of [chronic lower back pain] CLBP, we used multiple  qualitative research methods to characterize  within-team cross-referral and communication amongst jointly-trained practitioners representing diverse biomedical and complementary disciplines. (pg1) |
| Petersen, 2011(23) | South Africa | Qualitative design (Interviews) | “The aim of this study was to understand how the use  of the common implementation framework assisted in  the development of district/sub-district mental health  services in the two country contexts with the view to  drawing out shared lessons for integrating mental health into PHC in LMICs.” (pg3) |
| Rahmawati, 2015(24) | Indonesia | Qualitative design (Observation, Interviews) | “The objective of this study was to explore the role of a community-based program in supporting patients with hypertension in an Indonesian rural community.” (pg1) |
| Sada, 2011(25) | USA | Qualitative design (Interviews) | “To explore perceptions of primary care physicians’ (PCPs) and oncologists’ roles responsibilities, and patterns of communication related to shared cancer care in three integrated health systems that used electronic health records (EHRs).” (pg1) |
| Uittenbroek, 2018(26) | Netherlands | Qualitative design (Interviews) | “To explore how district nurses and social workers experience their new professional roles  as case managers within Embrace, a person-centered and integrated-care service for community-  living older adults.” (pg1) |
| Urtaran-Laresgoiti, 2018(27) | Spain | Qualitative design (Document review, Interviews) | A review of the strategy to challenge of chronicity in the Basque region of Spain. |
| Van Dongen, 2017(28) | Netherlands | Qualitative design (Observation, Interviews) | “This study aimed to improve our understanding of the process of interprofessional collaboration in primary care team meetings in the Netherlands by observing the current practice and exploring personal opinions.” (pg1) |
| Wakida, 2018(29) | Uganda | Qualitative design (Interviews) | “Using a case study approach [31, 32] and the Capability, Opportunity and Motivation framework for understanding behavior [33] the aim of this study was to explore the context specific factors affecting the ability of PCPs in rural Mbarara district to integrate mental health services into PHC.” (pg2) |
| Wakida, 2019(30) | Uganda | Qualitative design (Interviews) | “The purpose of this study was to explore the health systems constraints to the integration of mental health  services into PHC in Uganda from the perspective of primary health care providers (PHCPs).” (pg1) |
| Walters, 2012(31) | Netherlands | Qualitative design (Interviews) | The overall aim of the study is to explore  how disease management programs are implemented within primary care settings in the Netherlands; this paper focuses on the early development and implementation stages of five disease management programs in the primary are setting, based on interviews with project leadership teams.” (pg1) |
| Williams, 2019(32) | USA | Qualitative design (Interviews) | “We sought to gather opinions from primary care providers and administrators in Minnesota who were  involved in a CMS (Center for Medicare and Medicaid Services) transformational grant implementing COMPASS (Care Of Mental, Physical And Substance-use Syndromes), an evidence-based model of care coordination for depressed patients comorbid with diabetes and/or cardiovascular disease.” (pg1) |
| Wright, 2019(33) | USA | Qualitative design (Interviews) | “To describe the most effective model for managing, educating, and training pharmacist advanced clinical practitioners (ACPs) in the urgent care center (UCC) setting, role evolution and how to measure their effectiveness.” (pg1) |
| Zimbudzi, 2019(34) | Australia | Qualitative design (Focus groups, nterviews) | “In this study, we explored the experiences of patients and health-care providers, within this integrated diabetes and kidney service.” (pg1) |

**References**

1. Cramm JM, Tsiachristas A, Walters BH, Adams SA, Bal R, Huijsman R, et al. The management of cardiovascular disease in the Netherlands: analysis of different programmes. International Journal of Integrated Care [Electronic Resource]. 2013;13:e028.

2. Duner A, Blomberg S, Hasson H. Implementing a continuum of care model for older people-results from a Swedish case study. International Journal of Integrated Care [Electronic Resource]. 2011;11:e136.

3. Evans J, Grudniewicz A, Tsasis P. Trial and error, together: divergent thinking and collective learning inthe implementation of integrated care networks. International Review of Administrative Sciences. 2018; 84(3) 452–468.

4. Fitzpatrick SJ, Perkins D, Luland T, Brown D, Corvan E. The effect of context in rural mental health care: Understanding integrated services in a small town. Health & Place. 2017;45:70-6.

5. Grudniewicz A, Tenbensel T, Evans JM, Gray CS, Wodchis WP, Baker GR. Can complexity dynamics be harnessed to improve integration of care? The implementation of the Health Links in Ontario, Canada. International Journal of Integrated Care (IJIC). 2017;17(3):131-2.

6. Hayes S, Wolf C, Labbé S, Peterson E, Murray S. Primary health care providers' roles and responsibilities: A qualitative exploration of ‘ who does what ’ in the treatment and management of persons affected by obesity. Journal of Communication in Healthcare. 2017;10(1):47-54.

7. Henderson J, Dawson S, Fuller J, O'Kane D, Gerace A, Oster C, et al. Regional responses to the challenge of delivering integrated care to older people with mental health problems in rural Australia. Aging & Mental Health. 2018;22(8):1025-31.

8. Ingram M, Denman CA, Cornejo-Vucovich E, Castro-Vasquez MDC, Aceves B, Ocejo AG, et al. The Meta Salud Diabetes Implementation Study: Qualitative Methods to Assess Integration of a Health Promotion Intervention Into Primary Care to Reduce CVD Risk Among an Underserved Population With Diabetes in Sonora, Mexico. Frontiers in Public Health. 2019;7:347.

9. Jetelina KK, Woodson TT, Gunn R, Muller B, Clark KD, DeVoe JE, et al. Evaluation of an Electronic Health Record (EHR) Tool for Integrated Behavioral Health in Primary Care. Journal of the American Board of Family Medicine: JABFM. 2018;31(5):712-23.

10. Jones CD, Jones J, Richard A, Bowles K, Lahoff D, Boxer RS, et al. "Connecting the Dots": A Qualitative Study of Home Health Nurse Perspectives on Coordinating Care for Recently Discharged Patients. Journal of General Internal Medicine. 2017;32(10):1114-21.

11. Jones CD, Vu MB, O'Donnell CM, Anderson ME, Patel S, Wald HL, et al. A failure to communicate: a qualitative exploration of care coordination between hospitalists and primary care providers around patient hospitalizations. Journal of General Internal Medicine. 2015;30(4):417-24.

12. Kianfar S, Carayon P, Hundt AS, Hoonakker P. Care coordination for chronically ill patients: Identifying coordination activities and interdependencies. Applied Ergonomics. 2019;80:9-16.

13. Lalani M, Fernandes J, Fradgley R, Ogunsola C, Marshall M. Transforming community nursing services in the UK; lessons from a participatory evaluation of the implementation of a new community nursing model in East London based on the principles of the Dutch Buurtzorg model. BMC Health Services Research. 2019;19(1):945.

14. Leppin AL, Schaepe K, Egginton J, Dick S, Branda M, Christiansen L, et al. Integrating community-based health promotion programs and primary care: a mixed methods analysis of feasibility. BMC Health Services Research. 2018;18(1):72.

15. Limbani F, Thorogood M, Gomez-Olive FX, Kabudula C, Goudge J. Task shifting to improve the provision of integrated chronic care: realist evaluation of a lay health worker intervention in rural South Africa. BMJ Global Health. 2019;4(1):e001084.

16. Lindeman MA, Taylor KA, Kuipers P, Stothers K, Piper K. 'We don't have anyone with dementia here': a case for better intersectoral collaboration for remote Indigenous clients with dementia. Australian Journal of Rural Health. 2012;20(4):190-4.

17. Lockhart E, Hawker GA, Ivers NM, O'Brien T, Mukerji G, Pariser P, et al. Engaging primary care physicians in care coordination for patients with complex medical conditions. Canadian Family Physician. 2019;65(4):e155-e62.

18. Loeb DF, Bayliss EA, Candrian C, deGruy FV, Binswanger IA. Primary care providers' experiences caring for complex patients in primary care: a qualitative study. BMC Family Practice. 2016;17:34.

19. Lowe G, Plummer V, Boyd L. Nurse practitioner integration: Qualitative experiences of the change management process. Journal of Nursing Management (John Wiley & Sons, Inc). 2018;26(8):992-1001.

20. Mayer F, Bick D, Taylor C. Multidisciplinary care for pregnant women with cardiac disease: A mixed methods evaluation. International Journal of Nursing Studies. 2018;85:96-105.

21. Murphy J, Ko M, Kizer KW, Bindman AB. Safety net integration: a shared strategy for becoming providers of choice. Journal of Health Politics, Policy & Law. 2015;40(2):403-19.

22. O'Connor BB, Eisenberg DM, Buring JE, Liang CL, Osypiuk K, Levy DB, et al. Within-team Patterns of Communication and Referral in Multimodal Treatment of Chronic Low Back Pain Patients by an Integrative Care Team. Global Advances in Health & Medicine. 2015;4(2):36-45.

23. Petersen I, Ssebunnya J, Bhana A, Baillie K, Mha PPRPC. Lessons from case studies of integrating mental health into primary health care in South Africa and Uganda. International Journal of Mental Health Systems. 2011;5:8.

24. Rahmawati R, Bajorek B. A Community Health Worker-Based Program for Elderly People With Hypertension in Indonesia: A Qualitative Study, 2013. Preventing Chronic Disease. 2015;12:E175.

25. Sada YH, Street Jr RL, Singh H, Shada RE, Naik AD. Primary care and communication in shared cancer care: A qualitative study. American Journal of Managed Care. 2011;17(4):259-65.

26. Uittenbroek RJ, van der Mei SF, Slotman K, Reijneveld SA, Wynia K. Experiences of case managers in providing person-centered and integrated care based on the Chronic Care Model: A qualitative study on embrace. PLoS ONE [Electronic Resource]. 2018;13(11):e0207109.

27. Urtaran-Laresgoiti M, Álvarez-Rosete A, Nuño-Solinís R. A system-wide transformation towards integrated care in the Basque Country: A realist evaluation. International Journal of Care Coordination. 2018;21(3):98-108.

28. van Dongen JJ, van Bokhoven MA, Daniels R, Lenzen SA, van der Weijden T, Beurskens A. Interprofessional primary care team meetings: a qualitative approach comparing observations with personal opinions. Family Practice. 2017;34(1):98-106.

29. Wakida EK, Obua C, Rukundo GZ, Maling S, Talib ZM, Okello ES. Barriers and facilitators to the integration of mental health services into primary healthcare: a qualitative study among Ugandan primary care providers using the COM-B framework. BMC Health Services Research. 2018;18(1):890.

30. Wakida EK, Okello ES, Rukundo GZ, Akena D, Alele PE, Talib ZM, et al. Health system constraints in integrating mental health services into primary healthcare in rural Uganda: perspectives of primary care providers. International Journal of Mental Health Systems. 2019;13:16.

31. Walters BH, Adams SA, Nieboer AP, Bal R. Disease management projects and the Chronic Care Model in action: baseline qualitative research. BMC Health Services Research. 2012;12:114.

32. Williams MD, Asiedu GB, Finnie D, Neely C, Egginton J, Finney Rutten LJ, et al. Sustainable care coordination: a qualitative study of primary care provider, administrator, and insurer perspectives. BMC Health Services Research. 2019;19(1):92.

33. Wright DJ, Adams RJ, Blacklock J, Corlett SA, Harmston R, McWilliams M, et al. Longitudinal qualitative evaluation of pharmacist integration into the urgent care setting. Integrated Pharmacy Research & Practice. 2018;7:93-104.

34. Zimbudzi E, Lo C, Robinson T, Ranasinha S, Teede HJ, Usherwood T, et al. The impact of an integrated diabetes and kidney service on patients, primary and specialist health professionals in Australia: A qualitative study. PLoS ONE [Electronic Resource]. 2019;14(7):e0219685.
