# Supplementary material for: Implementation considerations for non-communicable disease-related integration in primary health care: a rapid review of qualitative evidence
Source: BMC Health Serv Res. 2023 Feb 18;23:169. doi: 10.1186/s12913-023-09151-x (PMC9938355; doi:10.1186/s12913-023-09151-x)
Supplement: Supplementary file 5 — Additional file 5: Table:CERQual assessments for seven main findings of the PHC Integration rapid review. [file 12913_2023_9151_MOESM5_ESM.docx]

**Additional file 5**

**Table: CERQual assessments for seven main findings of the PHC Integration rapid review**

| **Review finding** | **Overall CERQual assessment** | | **Data source** | **CASP assessment and setting of studies** | |
| --- | --- | --- | --- | --- | --- |
| **A: POLICY ALIGNMENT AND GOVERNANCE**  **Appropriate governance (political, legal, and administrative), that includes an enabling policy environment and policy alignment across management levels, as well as alignment with health system support services, are key to enable implementation and for sustainable integration reforms.** | **Moderate confidence** – it is likely that the review finding is a reasonable representation of the phenomenon of interest. (Downgraded due to minor concerns re methodological limitations, relevance, and adequacy). | | (1-18) | 1. Montenegro et al. 2011. CASP – moderate concerns (Argentina, Belize, Brazil, Chile, Cuba, Ecuador, Mexico, Paraguay, Trinidad and Tobago, and Uruguay, Central America, and Eastern Caribbean) 2. Mayhew et al. 2017. CASP – no concerns (Kenya) 3. McIntosh et al. 2017. CASP – no concerns (Germany, Greece, Italy, Northern Ireland, Portland, Portugal, Scotland, Spain, Sweden) 4. Petersen et al. 2019. CASP – no concerns (India, Nepal, Ethiopia, Nigeria, South Africa, Uganda) 5. Hosey et al. 2016. CASP – moderate concerns (6 US Associated Pacific Islands) 6. Stadnick et al. 2019. CASP – no to minor concerns Stadnick et al. 2019. CASP – no to minor concerns (North & South American, Europe, Asia, African countries) 7. Kozlowska et al. 2020. CASP – no or very minor concerns (Europe and UK) 8. Venables et al. 2016. CASP – no to very minor concerns (Kenya) 9. Lovero et al. 2019. CASP – moderate concerns (South Africa) 10. Ignatowicz et al. 2014. CASP – no concerns (Europe & UK) 11. Kalonji et al. 2019. CASP – moderate to serious concerns (South Africa) 12. Lupafya et al. 2016. CASP – moderate to serious concerns (Malawi) 13. Schmidt et al. 2016. CASP – no to minor concerns (Australia)   14. Duffy et al. 2017. CASP - minor concerns (Zambia, Kenya, Uganda, and Cambodia)  15. Steele Gray et al. 2018. CASP – no to minor concerns (Canada and New Zealand)  16. Zou et al. 2020. CASP – no concerns (China)  17. Fuller et al. 2015. CASP – no to minor concerns (Australia)  18. Lawn et al. 2014. CASP – no concerns (Australia) | |
|  | | **CERQual assessment – components** | | | |
| **Methodological limitations** | **Coherence** | |  | **Relevance** | **Adequacy** |
| **Minor concerns** regarding the methodological limitations because only one study had moderate to serious concerns | **No concerns** regarding coherence as good fit between the finding and the underlying data. | |  | **Minor concerns** regarding relevance. Fair applicability of underlying evidence to the review question due to diversity of settings, but fewer sources focused explicitly on NCD-CD integration. | **Minor concerns** regarding adequacy due to fair number of contributing studies, though thin data and partial contribution of individual studies reduced adequacy. |
| **Review finding** | **Overall CERQual assessment** | | **Data source** | **CASP assessment and setting of studies** | |
| **B: HEALTH SYSTEMS READINESS, INTERVENTION COMPATIBILITY AND LEADERSHIP**  **Health system readiness for change, compatibility of the new integration intervention with existing organisational practices, and the quality of leadership are key factors that can influence the success of integration.** | **Moderate confidence** - it is likely that the review finding is a reasonable representation of the phenomenon of interest. (Downgraded due to minor concerns re methodological limitations, relevance, and adequacy). | | (1-20) | 1. Montenegro et al. 2011. CASP – moderate concerns (Argentina, Belize, Brazil, Chile, Cuba, Ecuador, Mexico, Paraguay, Trinidad and Tobago, and Uruguay, Central America, and Eastern Caribbean) 2. Mayhew et al. 2017. CASP – no concerns (Kenya) 3. McIntosh et al. 2017. CASP – no concerns (Germany, Greece, Italy, Northern Ireland, Portland, Portugal, Scotland, Spain, Sweden) 4. Petersen et al. 2019. CASP – no concerns (India, Nepal, Ethiopia, Nigeria, South Africa, Uganda) 5. Hosey et al. 2016. CASP – moderate concerns (6 US Associated Pacific Islands) 6. Stadnick et al. 2019. CASP – no to minor concerns Stadnick et al. 2019. CASP – no to minor concerns (North & South American, Europe, Asia, African countries) 7. Kozlowska et al. 2020. CASP – no or very minor concerns (Europe and UK) 8. Venables et al. 2016. CASP – no to very minor concerns (Kenya) 9. Lovero et al. 2019. CASP – moderate concerns (South Africa) 10. Ignatowicz et al. 2014. CASP – no concerns (Europe & UK) 11. Kalonji et al. 2019. CASP – moderate to serious concerns (South Africa) 12. Lupafya et al. 2016. CASP – moderate to serious concerns (Malawi)   13. Schmidt et al. 2016. CASP – no to minor concerns (Australia)  14. Duffy et al. 2017. CASP - minor concerns (Zambia, Kenya, Uganda, and Cambodia)  15. Steele Gray et al. 2018. CASP – no to minor concerns (Canada and New Zealand)  16. Zou et al. 2020. CASP – no concerns (China)  17. Fuller et al. 2015. CASP – no to minor concerns (Australia)  18. Lawn et al. 2014. CASP – no concerns (Australia)  19. Shelley et al. 2019. CASP – minor concerns (Tanzania)  20. White et al. 2013. CASP – no to minor concerns  (Cambodia) | |
|  | | **CERQual assessment – components** | | | |
| **Methodological limitations** | **Coherence** | |  | **Relevance** | **Adequacy** |
| **Minor concerns** regarding the methodological limitations because only two studies had moderate methodological concerns | **No concerns** regarding coherence as good fit between the finding and the underlying data. | |  | **Minor concerns** regarding relevance. Fair applicability of underlying evidence to the review question due to diversity of settings, but fewer sources focused explicitly on NCD-CD integration. | **Minor concerns** regarding adequacy due to the fair number of contributing studies, though thin data and partial contribution of individual studies reduced adequacy. |
| **Review finding** | **Overall CERQual assessment** | | **Data source** | **CASP assessment and setting of studies** | |
| **C: HUMAN RESOURCE MANAGEMENT, DEVELOPMENT AND SUPPORT**  **Human resource management, development and support are major considerations for implementing integration of services, with the need for strategic realignment of financial, management, and human resource and supervision systems, in support of the integration reform objectives. This involves human resource availability, appropriate skills, and training of staff, as well as supportive management and supervision.** | **Moderate confidence** - it is likely that the review finding is a reasonable representation of the phenomenon of interest (Downgraded due to minor concerns re methodological limitations, relevance, and minor concerns re adequacy). | | (2-6, 8, 9, 11-17, 19, 20) | 2. Mayhew et al. 2017. CASP – no concerns (Kenya)  3. McIntosh et al. 2017. CASP – no concerns (Germany, Greece, Italy, Northern Ireland, Portland, Portugal, Scotland, Spain, Sweden)  4. Petersen et al. 2019. CASP – no concerns (India, Nepal, Ethiopia, Nigeria, South Africa, Uganda)  5. Hosey et al. 2016. CASP – moderate concerns (6 US Associated Pacific Islands)  6. Stadnick et al. 2019. CASP – no to minor concerns Stadnick et al. 2019. CASP – no to minor concerns (North & South American, Europe, Asia, African countries)  8. Venables et al. 2016. CASP – no to very minor concerns (Kenya)  9. Lovero et al. 2019. CASP – moderate concerns (South Africa)  11. Kalonji et al. 2019. CASP – moderate to serious concerns (South Africa)  12. Lupafya et al. 2016. CASP – moderate to serious concerns (Malawi)  13. Schmidt et al. 2016. CASP – no to minor concerns (Australia)  14. Duffy et al. 2017. CASP - minor concerns (Zambia, Kenya, Uganda, and Cambodia)  15. Steele Gray et al. 2018. CASP – no to minor concerns (Canada and New Zealand)  16. Zou et al. 2020. CASP – no concerns (China)  17. Fuller et al. 2015. CASP – no to minor concerns (Australia)  19. Shelley et al. 2019. CASP – minor concerns (Tanzania)  20. White et al. 2013. CASP – no to minor concerns  (Cambodia) | |
|  | | **CERQual assessment - components** | | | |
| **Methodological limitations** | **Coherence** | |  | **Relevance** | **Adequacy** |
| **Minor concerns** regarding the methodological limitations because only one study had moderate to serious concerns. | **No concerns** regarding coherence as good fit between the finding and the underlying data. | |  | **Minor concerns** regarding relevance. Fair applicability of underlying evidence to the review question due to diversity of settings, but fewer sources focused explicitly on NCD-CD integration. | **Very minor concerns** regarding adequacy due to the fair number of contributing studies, though thin date and partial contribution of individual studies reduced adequacy. |

**References**

1. Montenegro H, Holder R, Ramagem C, Urrutia S, Fabrega R, Tasca R, et al. Combating health care fragmentation through integrated health service delivery networks in the Americas: lessons learned. Journal of Integrated Care. 2011;19(5):5-16.

2. Mayhew SH, Sweeney S, Warren CE, Collumbien M, Ndwiga C, Mutemwa R, et al. Numbers, systems, people: how interactions influence integration. Insights from case studies of HIV and reproductive health services delivery in Kenya. Health Policy & Planning. 2017;32(suppl_4):iv67-iv81.

3. McIntosh J, Alonso A, Codina C, MacLure K, Mair A, Stewart D. Polypharmacy and Integrated Care: Identifying synergies in implementation. International Journal of Integrated Care (IJIC). 2017;17:1-2.

4. Petersen I, van Rensburg A, Kigozi F, Semrau M, Hanlon C, Abdulmalik J, et al. Scaling up integrated primary mental health in six low- and middle-income countries: obstacles, synergies and implications for systems reform. BJPsych Open. 2019;5(5):e69.

5. Hosey GM, Rengiil A, Maddison R, Agapito AU, Lippwe K, Wally OD, et al. U.S. Associated Pacific Islands Health Care Teams Chart a Course for Improved Health Systems: Implementation and Evaluation of a Non-communicable Disease Collaborative Model. J Health Care Poor Underserved. 2016;27(4A):19-38.

6. Stadnick NA, Sadler E, Sandall J, Turienzo CF, Bennett IM, Borkan J, et al. Comparative case studies in integrated care implementation from across the globe: a quest for action. BMC Health Services Research. 2019;19(1):899.

7. Kozlowska O, Seda Gombau G, Rea R. Leadership for integrated care: a case study. Leadership in Health Services. 2020.

8. Venables E, Edwards JK, Baert S, Etienne W, Khabala K, Bygrave H. "They just come, pick and go." The Acceptability of Integrated Medication Adherence Clubs for HIV and Non Communicable Disease (NCD) Patients in Kibera, Kenya. PLoS ONE [Electronic Resource]. 2016;11(10):e0164634.

9. Lovero KL, Lammie SL, van Zyl A, Paul SN, Ngwepe P, Mootz JJ, et al. Mixed-methods evaluation of mental healthcare integration into tuberculosis and maternal-child healthcare services of four South African districts. BMC Health Services Research. 2019;19(1):83.

10. Ignatowicz A, Greenfield G, Pappas Y, Car J, Majeed A, Harris M. Achieving provider engagement: providers' perceptions of implementing and delivering integrated care. Qualitative Health Research. 2014;24(12):1711-20.

11. Kalonji D, Mahomed OH. Health system challenges affecting HIV and tuberculosis integration at primary healthcare clinics in Durban, South Africa. African Journal of Primary Health Care & Family Medicine. 2019;11(1):e1-e7.

12. Lupafya PC, Mwagomba BL, Hosig K, Maseko LM, Chimbali H. Implementation of Policies and Strategies for Control of Noncommunicable Diseases in Malawi: Challenges and Opportunities. Health Education & Behavior. 2016;43(1 Suppl):64S-9S.

13. Schmidt B, Campbell S, McDermott R. Community health workers as chronic care coordinators: evaluation of an Australian Indigenous primary health care program. Australian & New Zealand Journal of Public Health. 2016;40 Suppl 1:S107-14.

14. Duffy M, Ojikutu B, Andrian S, Sohng E, Minior T, Hirschhorn LR. Non-communicable diseases and HIV care and treatment: models of integrated service delivery. Tropical Medicine & International Health. 2017;22(8):926-37.

15. Steele Gray C, Barnsley J, Gagnon D, Belzile L, Kenealy T, Shaw J, et al. Using information communication technology in models of integrated community-based primary health care: learning from the iCOACH case studies. Implementation Science. 2018;13(1):87.

16. Zou G, Zhang W, King R, Zhang Z, Walley J, Gong W, et al. Process Evaluation of a Clustered Randomized Control Trial of a Comprehensive Intervention to Reduce the Risk of Cardiovascular Events in Primary Health Care in Rural China. Int J Environ Res Public Health. 2020;17(11).

17. Fuller J, Koehne K, Verrall CC, Szabo N, Bollen C, Parker S. Building chronic disease management capacity in General Practice: The South Australian GP Plus Practice Nurse Initiative. Collegian: Journal of the Royal College of Nursing, Australia. 2015;22(2):191-7.

18. Lawn S, Lloyd A, King A, Sweet L, Gum L. Integration of primary health services: being put together does not mean they will work together. BMC Research Notes. 2014;7:66.

19. Shelley KD, Frumence G, Mpembeni R, Mwinnyaa G, Joachim J, Kisusi HK, et al. "Because Even the Person Living With HIV/AIDS Might Need to Make Babies" - Perspectives on the Drivers of Feasibility and Acceptability of an Integrated Community Health Worker Model in Iringa, Tanzania. International Journal of Health Policy & Management. 2019;8(9):538-49.

20. White J, Delvaux T, Chhea C, Saramony S, Ouk V, Saphonn V. The Linked Response: Lessons Emerging from Integration of HIV and Reproductive Health Services in Cambodia. AIDS Research & Treatment. 2013;2013:504792.
